# Supplementary material for: Unveiling relationships between crime and property in England and Wales via density scale-adjusted metrics and network tools
Source: PLoS One. 2018 Feb 22;13(2):e0192931. doi: 10.1371/journal.pone.0192931 (PMC5823401; doi:10.1371/journal.pone.0192931)
Supplement: S1 File — (PDF) [file pone.0192931.s002.pdf]

ASB

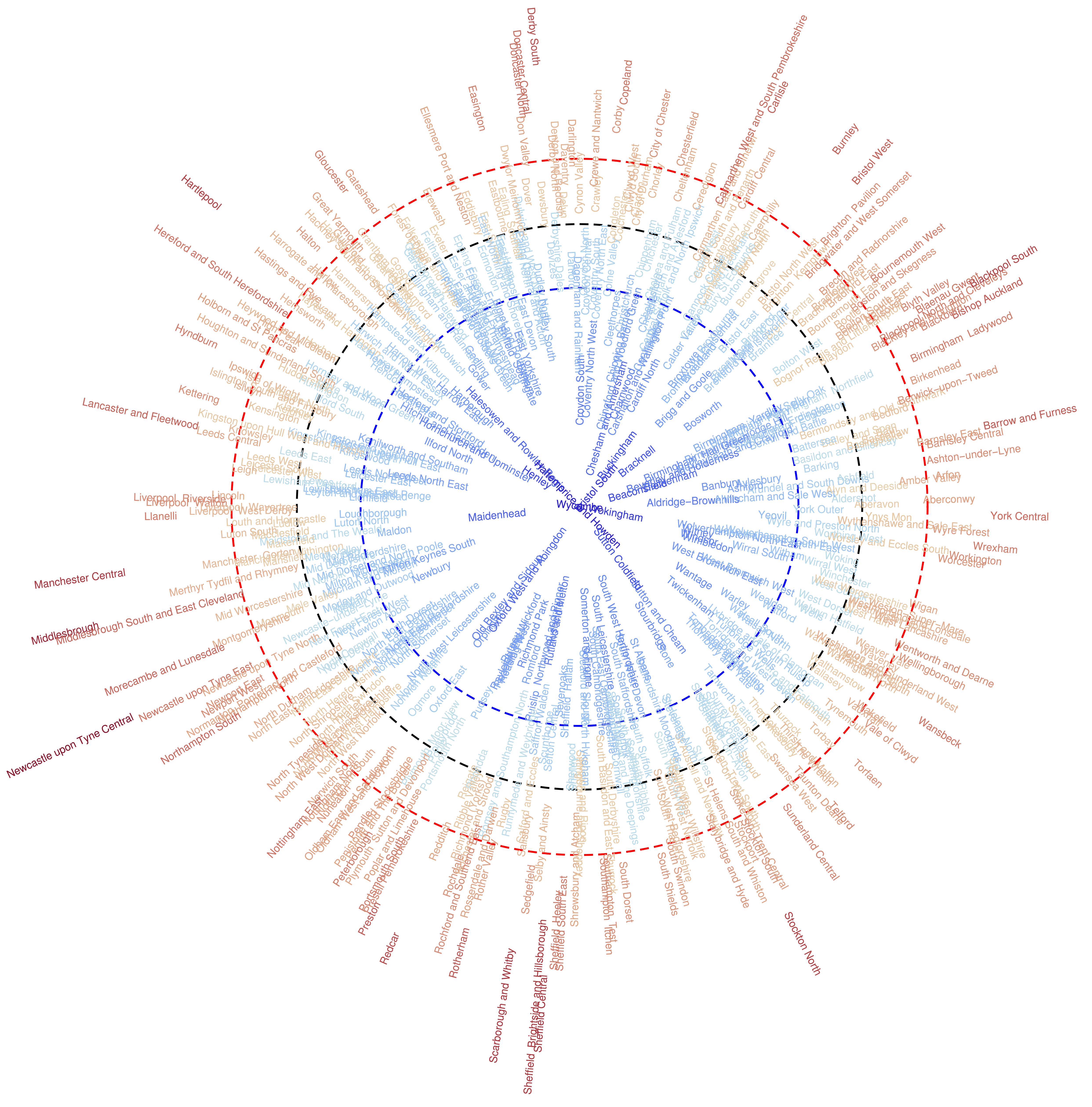

Derby South
Easington
Hartlepool
Gloucester
Gateshead
Hereford and South Herefordshire
Hyndburn
Kettering
Lancaster and Fleetwood
Leeds Central
Liverpool, Riverside
Llanelli
Manchester Central
Middlesbrough
Middlesbrough South and East Cleveland
Morecambe and Lunesdale
Newcastle upon Tyne Central
Redcar
Rotherham
Scarborough and Whitby
Stockton North
Sunderland Central
Wansbeck
Torfaen
Telford
Wrexham
Worcester
York Central
Barrow and Furness
Bishop Auckland
Blackpool South
Burnley
Bristol West
Carlisle
Chesterfield
Birmingham, Ladywood
Maidenhead
Wokingham
Sutton Coldfield
Aldridge−Brownhills

Bike Theft

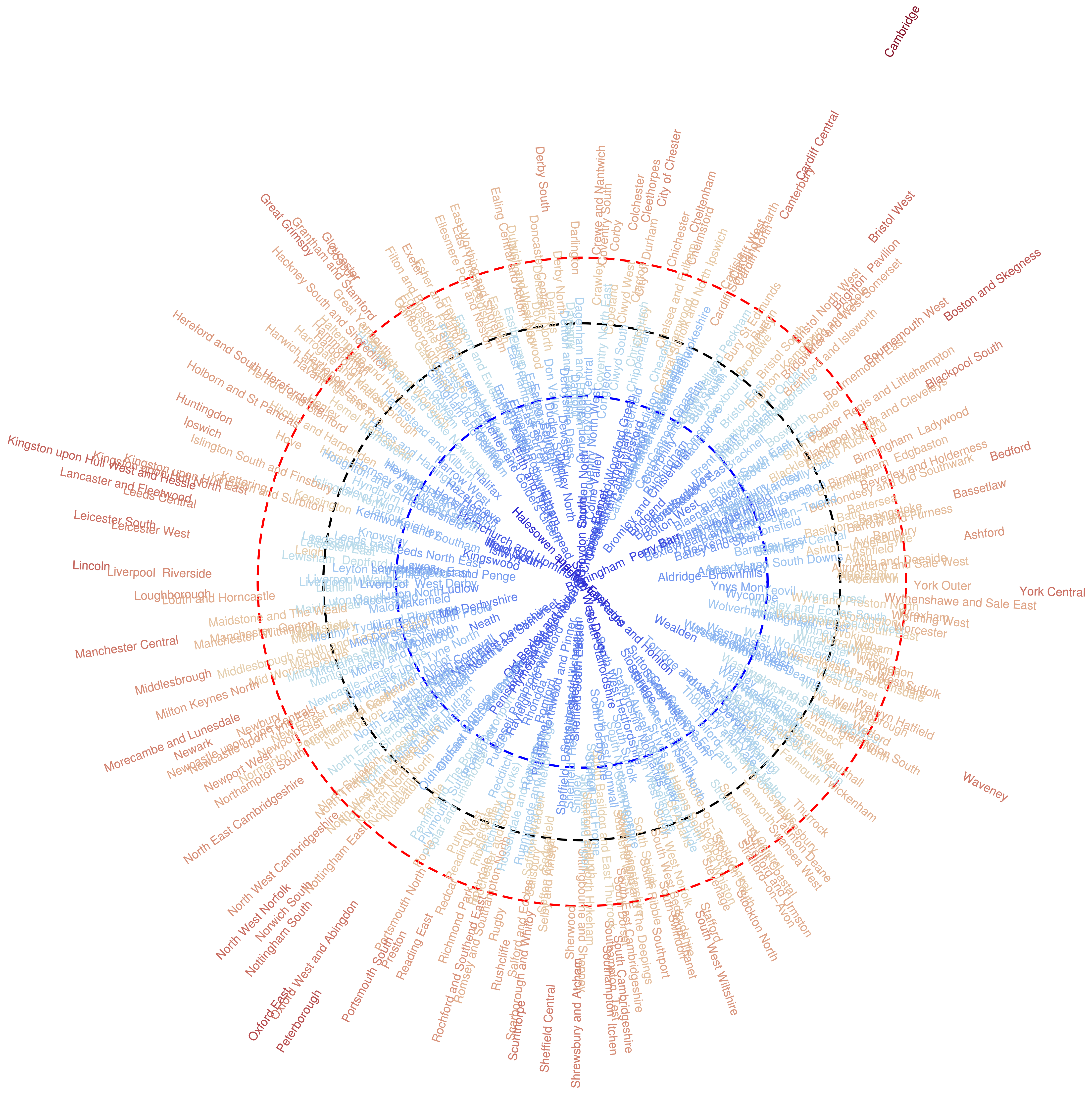
Cambridge
Cardiff Central
Canterbury
Bristol West
Boston and Skegness
Bournemouth West
Blackpool South
Bedford
Bassetlaw
Ashford
York Central
York Outer
Waveney
Kingston upon Hull West and Hessle
Lancaster and Fleetwood
Leeds Central
Leicester South
Leicester West
Lincoln
Liverpool Riverside
Loughborough
Manchester Central
Middlesbrough
Milton Keynes North
Morecambe and Lunesdale
Newark
North East Cambridgeshire
North West Cambridgeshire
North West Norfolk
Norwich South
Nottingham South
Oxford East
Oxford West and Abingdon
Peterborough
Portsmouth South
Preston
Reading East
Rushcliffe
Scunthorpe
Sheffield Central
Shrewsbury and Atcham
Southampton Itchen
South Cambridgeshire
South West Wiltshire
Stockton North
Derby South
Colchester
Cleethorpes
City of Chester
Cheltenham
Chelmsford
Great Grimsby
Gloucester
Hereford and South Herefordshire
Holborn and St Pancras
Huntingdon
Ipswich
Islington South and Finsbury

Burglary

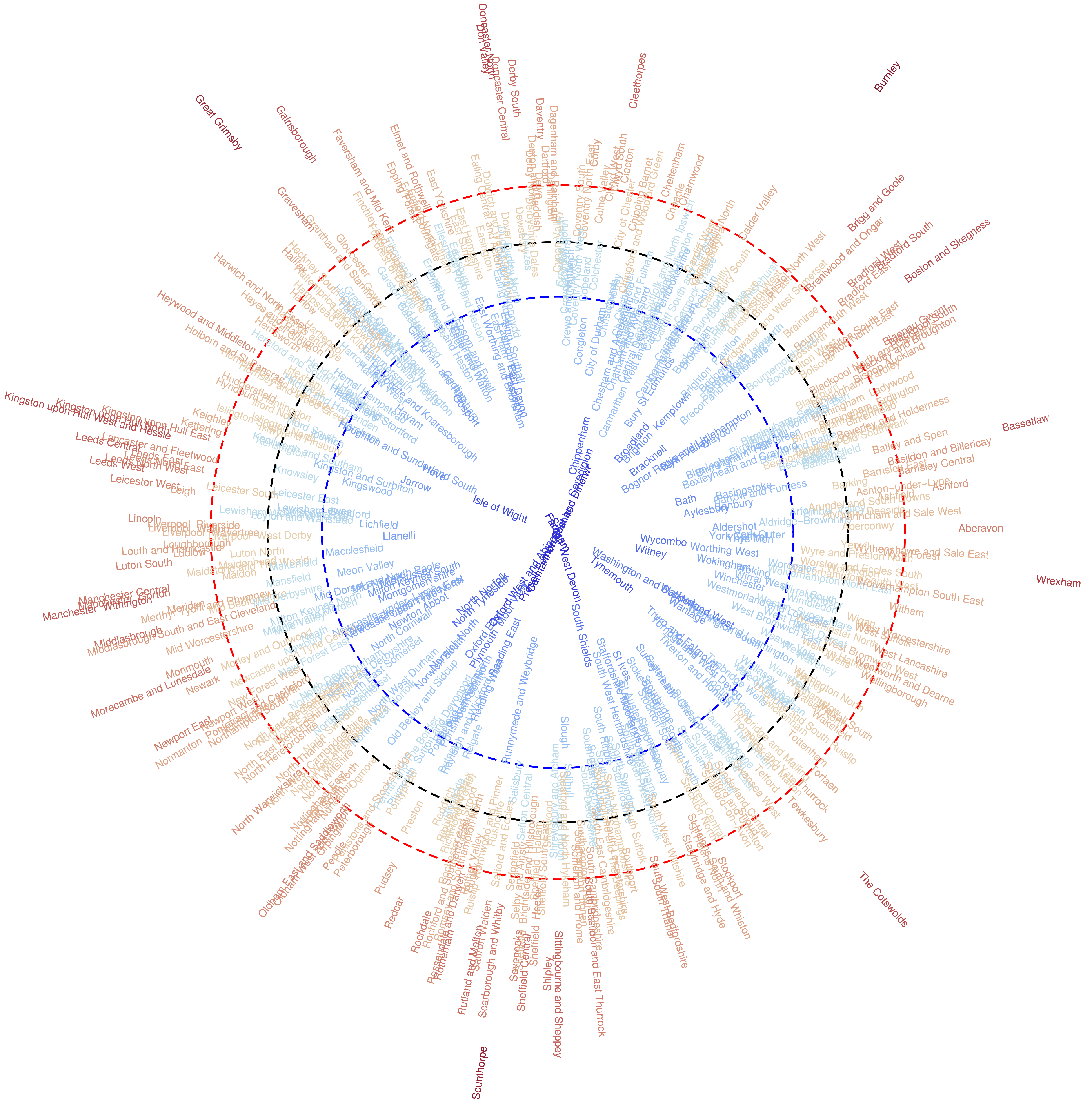
Burnley
Cleethorpes
Great Grimsby
Gainsborough
Derby South
Doncaster Central
Brigg and Goole
Boston and Skegness
Bradford South
Bassetlaw
Aberavon
Wrexham
Kingston upon Hull West and Hessle
Leeds Central
Leicester West
Lincoln
Luton South
Manchester Central
Middlesbrough
Morecambe and Lunesdale
Newport East
Normanton
Oldham East and Saddleworth
Pudsey
Redcar
Scunthorpe
Sittingbourne and Sheppey
Rutland and Melton
Scarborough and Whitby
Stockport
The Cotswolds
Tewkesbury
Wellingborough
Witham
Isle of Wight

CD and A

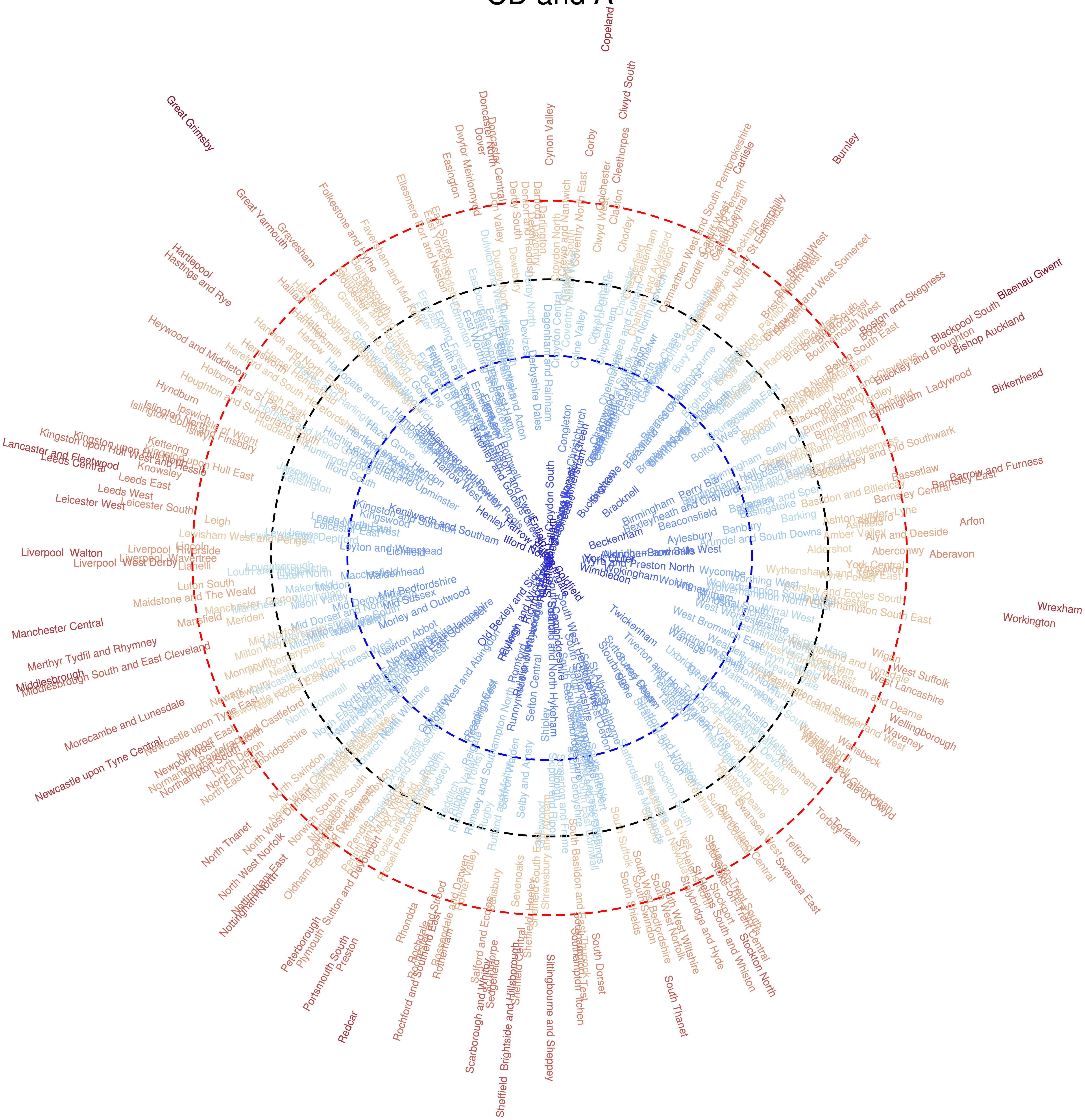

Copeland
Clwyd South
Great Grimsby
Burnley
Blaenau Gwent
Blackpool South
Bishop Auckland
Birkenhead
Barrow and Furness
Arfon
Aberavon
Wrexham
Workington
West Suffolk
West Lancashire
Wellingborough
Torfaen
Telford
Swansea East
Stockton North
South Thanet
South Dorset
Sittingbourne and Sheppey
Sheffield Brightside and Hillsborough
Scarborough and Whitby
Redcar
Portsmouth South
Preston
Peterborough
Nottingham North
North Thanet
Newcastle upon Tyne Central
Morecambe and Lunesdale
Middlesbrough
Middlesbrough South and East Cleveland
Merthyr Tydfil and Rhymney
Manchester Central
Liverpool Walton
Leicester West
Leeds Central
Lancaster and Fleetwood
Hartlepool
Hastings and Rye
Great Yarmouth
Gravesham
Heywood and Middleton
Hyndburn
Carlisle
Caerphilly
Cleethorpes
Corby
Cynon Valley
Dover
Easington

Detached

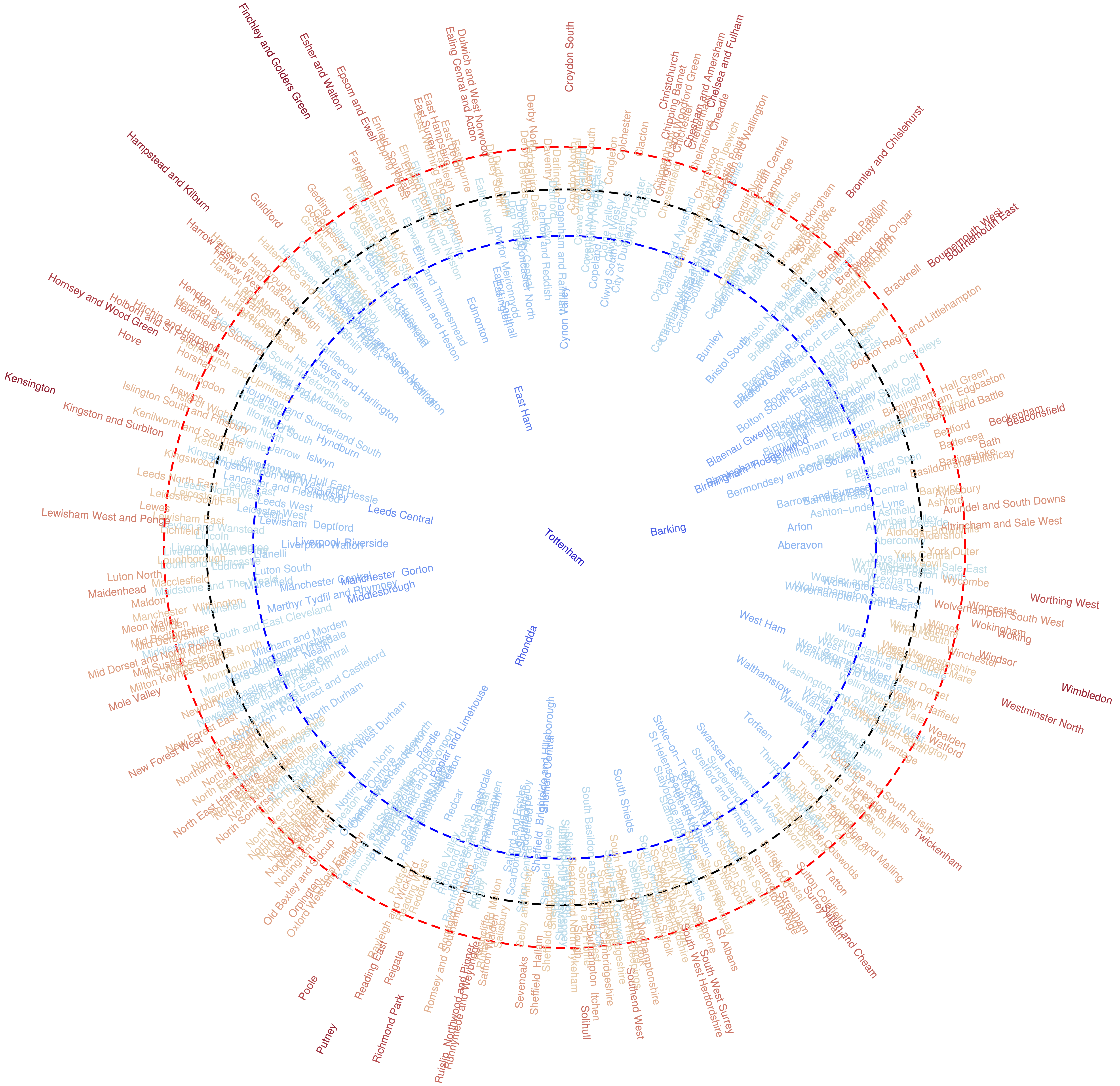

Finchley and Golders Green
Esher and Walton
Epsom and Ewell
Hampstead and Kilburn
Croydon South
Chelsea and Fulham
Chesham and Amersham
Bromley and Chislehurst
Bournemouth East
Bournemouth West
Beaconsfield
Beckenham
Bracknell
Arundel and South Downs
Altrincham and Sale West
Worthing West
Wimbledon
Westminster North
Windsor
Woking
Wokingham
Watford
Wealden
Twickenham
Tatton
Sutton and Cheam
South West Surrey
South West Hertfordshire
Southend West
Solihull
Sevenoaks
Ruislip, Northwood and Pinner
Richmond Park
Putney
Poole
Reigate
Reading East
Orpington
Old Bexley and Sidcup
New Forest West
Mole Valley
Mid Dorset and North Poole
Maidenhead
Luton North
Lewisham West and Penge
Kingston and Surbiton
Kensington
Hove
Hornsey and Wood Green
Holborn and St Pancras
Harrow East
Guildford
Tottenham
Barking
East Ham
Rhondda
West Ham
Walthamstow
Torfaen
South Shields
Leeds Central
Edmonton
Cynon Valley
Middlesbrough
Manchester Gorton
Liverpool Riverside
Deptford
Islwyn
Hyndburn
Arfon
Aberavon
Burnley
Bristol South
Redcar

Drugs

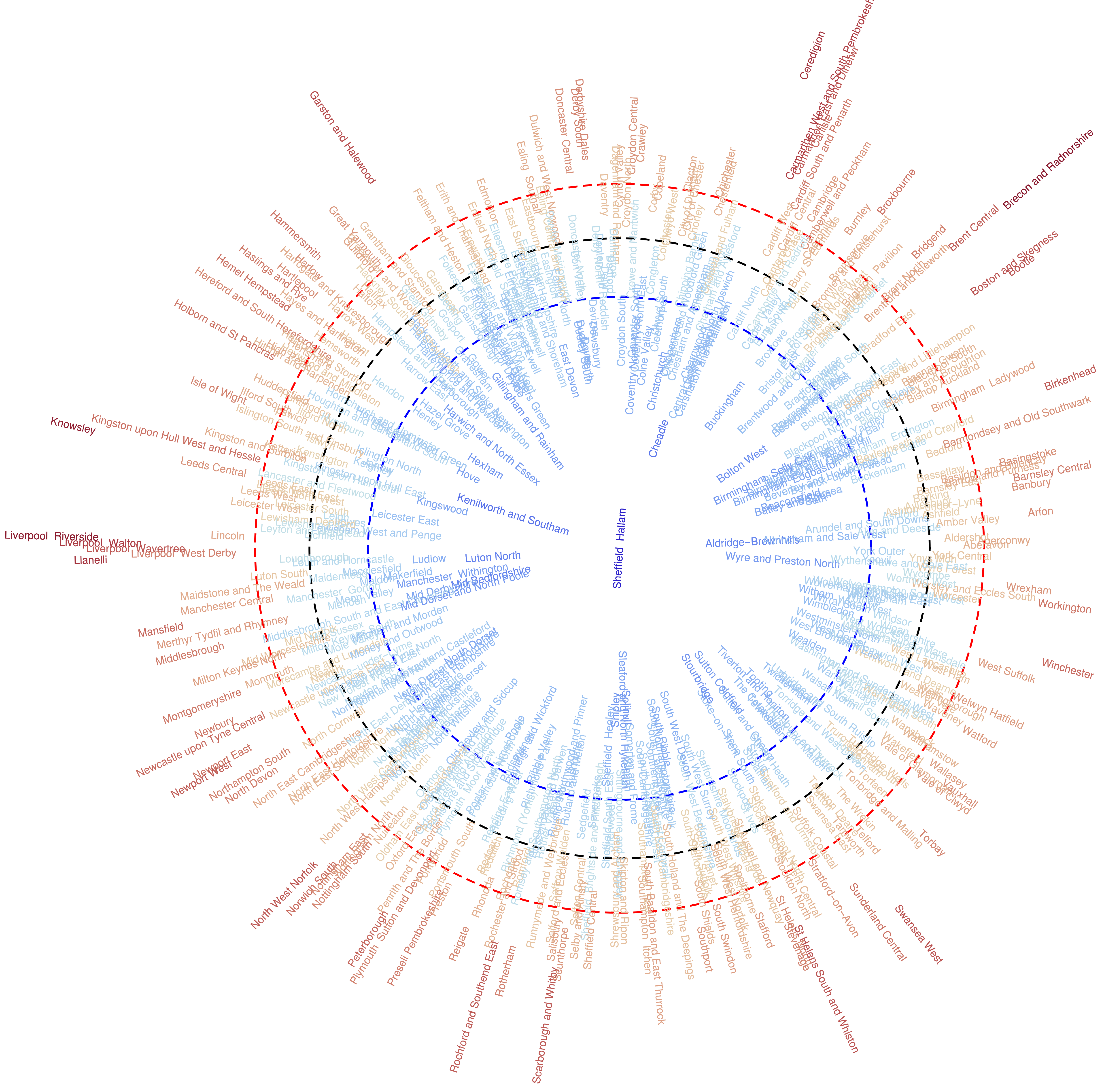

Ceredigion
Carmarthen West and South Pembrokeshire
Brecon and Radnorshire
Brent Central
Boston and Skegness
Bootle
Birkenhead
Bermondsey and Old Southwark
Barnsley Central
Banbury
Arfon
Wrexham
Workington
Winchester
West Suffolk
Welwyn Hatfield
Torbay
Swansea West
Sunderland Central
St Helens South and Whiston
Scarborough and Whitby
Rochford and Southend East
Rotherham
Reigate
Preseli Pembrokeshire
Plymouth Sutton and Devonport
Peterborough
North West Norfolk
Norwich South
Nottingham South
Newport West
Newport East
Newcastle upon Tyne Central
Newbury
Montgomeryshire
Milton Keynes North
Middlesbrough
Merthyr Tydfil and Rhymney
Mansfield
Manchester Central
Maidstone and The Weald
Liverpool Riverside
Liverpool Walton
Liverpool Wavertree
Liverpool West Derby
Llanelli
Lincoln
Leeds Central
Kingston upon Hull West and Hessle
Knowsley
Isle of Wight
Holborn and St Pancras
Hereford and South Herefordshire
Hemel Hempstead
Hastings and Rye
Hammersmith
Garston and Halewood
Doncaster Central
Derbyshire Dales
Sheffield Hallam
Kenilworth and Southam
Aldridge-Brownhills
Wyre and Preston North
Bolton West
Cheadle
Buckingham

Flats

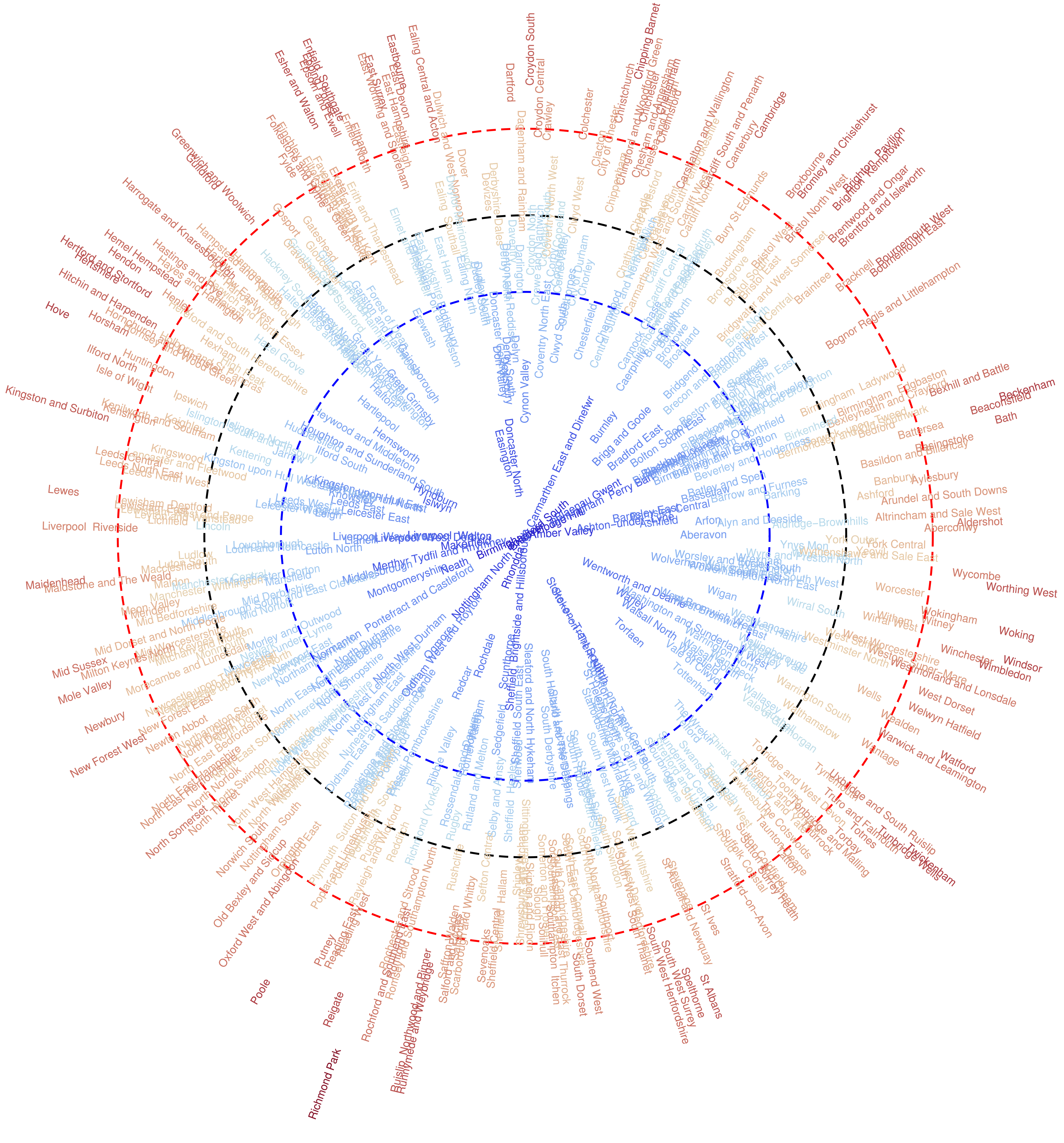

Croydon South
Dartford
Croydon Central
Crawley
Colchester
Chipping Barnet
Christchurch
Cambridge
Canterbury
Bury St Edmunds
Broxbourne
Bromley and Chislehurst
Brighton Pavilion
Brighton Kemptown
Brentwood and Ongar
Brentford and Isleworth
Bournemouth West
Bournemouth East
Bracknell
Braintree
Bognor Regis and Littlehampton
Bexhill and Battle
Beckenham
Beaconsfield
Bath
Battersea
Basingstoke
Basildon and Billericay
Banbury
Aylesbury
Ashford
Arundel and South Downs
Altrincham and Sale West
Aldershot
Aberconwy
York Outer
York Central
Wycombe
Worthing West
Worcester
Wokingham
Woking
Witney
Witham
Windsor
Winchester
Wimbledon
Westmorland and Lonsdale
West Dorset
Wells
Welwyn Hatfield
Wealden
Watford
Warwick and Leamington
Wantage
Uxbridge and South Ruislip
Twickenham
Tunbridge Wells
Truro and Falmouth
Totnes
Torbay
St Ives
St Albans
Spelthorne
South West Surrey
South West Hertfordshire
South West Norfolk
Southend West
South Dorset
Stratford-on-Avon
Richmond Park
Reigate
Poole
Putney
Oxford West and Abingdon
Old Bexley and Sidcup
Norwich South
North Somerset
New Forest West
Newbury
Mole Valley
Mid Sussex
Maidenhead
Liverpool Riverside
Lewes
Kingston and Surbiton
Hove
Hitchin and Harpenden
Hertsmere
Hertford and Stortford
Hendon
Hemel Hempstead
Harrogate and Knaresborough
Greenwich and Woolwich
Guildford
Esher and Walton
Epsom and Ewell
Enfield Southgate
Ealing Central and Acton
Eastbourne
East Worthing and Shoreham
Isle of Wight
Ilford North
Horsham
Ipswich
Runnymede and Weybridge
Ruislip, Northwood and Pinner
Rochford and Southend East

Freehold

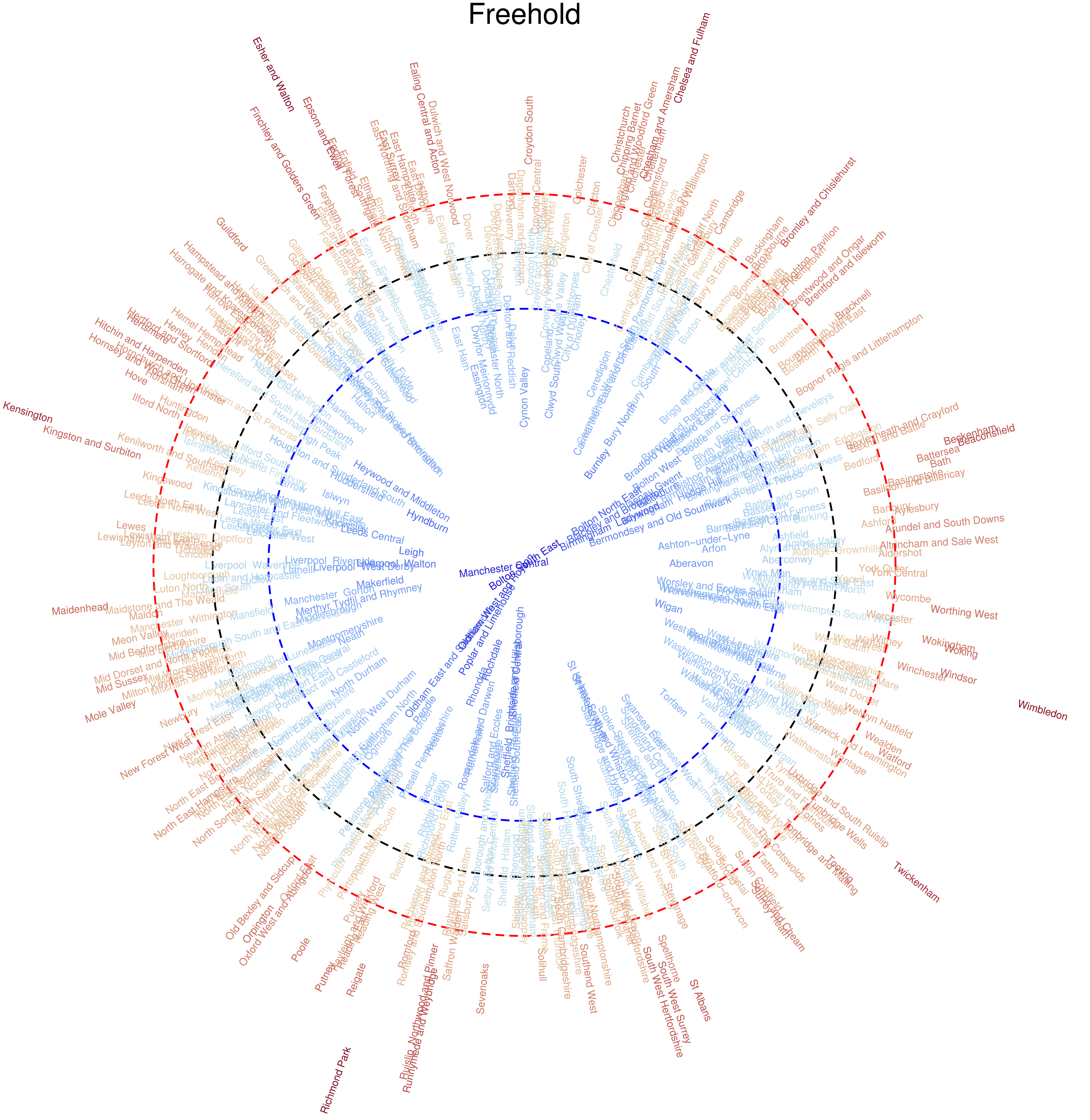
Esher and Walton
Chelsea and Fulham
Croydon South
Epsom and Ewell
Finchley and Golders Green
Ealing Central and Acton
Dulwich and West Norwood
Guildford
Bromley and Chislehurst
Brentwood and Ongar
Brentford and Isleworth
Bognor Regis and Littlehampton
Bexleyheath and Crayford
Beckenham
Beaconsfield
Battersea
Bath
Basildon and Billericay
Aylesbury
Arundel and South Downs
Altrincham and Sale West
York Central
Wycombe
Worthing West
Wokingham
Woking
Winchester
Windsor
Wimbledon
Welwyn Hatfield
Warwick and Leamington
Watford
Wealden
Uxbridge and South Ruislip
Tunbridge Wells
Tonbridge and Malling
Tooting
Twickenham
The Cotswolds
Sutton and Cheam
Stratford-on-Avon
St Albans
South West Surrey
South West Hertfordshire
Spelthorne
Southend West
Solihull
Sevenoaks
Saffron Walden
Ruislip, Northwood and Pinner
Runnymede and Weybridge
Richmond Park
Reigate
Putney
Poole
Old Bexley and Sidcup
Orpington
Oxford West and Abingdon
New Forest West
Newbury
Mole Valley
Mid Sussex
Mid Dorset and North Poole
Maidenhead
Maidstone and The Weald
Lewes
Leeds North East
Kingswood
Kenilworth and Southam
Kingston and Surbiton
Kensington
Ilford North
Hove
Hornsey and Wood Green
Hitchin and Harpenden
Hertford and Stortford
Hemel Hempstead
Harrogate and Knaresborough
Hampstead and Kilburn
Manchester Central
Cynon Valley
Hyndburn
Leigh
Wigan
Torfaen
Aberavon
Arfon
Ashton-under-Lyne
Heywood and Middleton
Rhondda
Rochdale

Leasehold

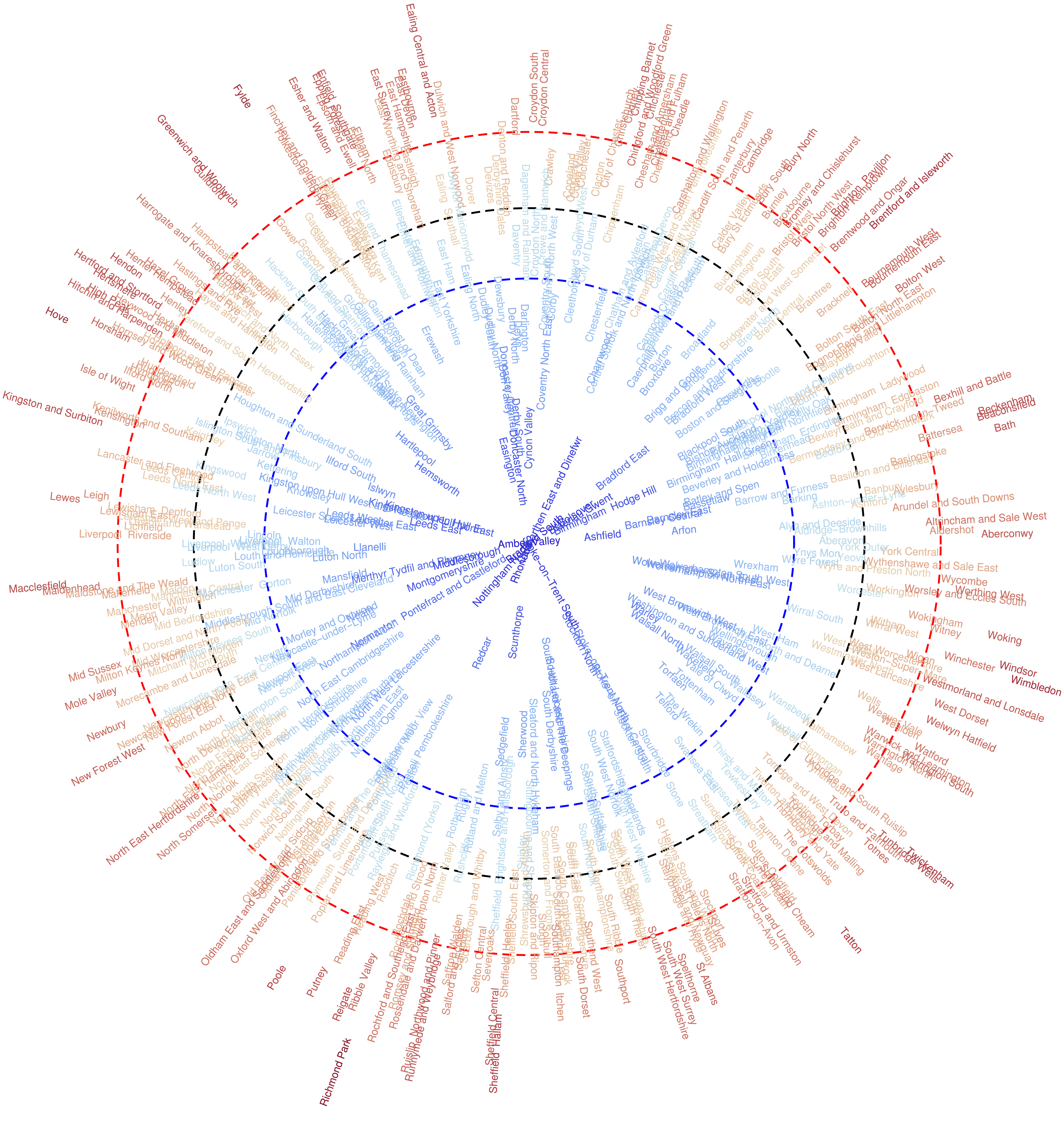
Ealing Central and Acton
Croydon South
Croydon Central
Fylde
Greenwich and Woolwich
Esher and Walton
Hove
Horsham
Isle of Wight
Kingston and Surbiton
Lewes
Leigh
Liverpool Riverside
Macclesfield
Mid Sussex
Mole Valley
Newbury
New Forest West
North East Hertfordshire
North Somerset
Oldham East and Saddleworth
Oxford West and Abingdon
Poole
Putney
Reigate
Ribble Valley
Richmond Park
Sheffield Hallam
Sheffield Central
South West Hertfordshire
Spelthorne
South West Surrey
St Albans
Tatton
Twickenham
Tunbridge Wells
Totnes
Wimbledon
Windsor
Woking
Westmorland and Lonsdale
West Dorset
Welwyn Hatfield
Worthing West
Wycombe
Aberconwy
Altrincham and Sale West
Arundel and South Downs
Aldershot
Bath
Beckenham
Beaconsfield
Battersea
Bexhill and Battle
Brentford and Isleworth
Brentwood and Ongar
Bromley and Chislehurst
Bury North
Cambridge
Canterbury
Carshalton and Wallington
Chipping Barnet
Dartford
Hendon
Hertsmere
Hitchin and Harpenden
Hertford and Stortford
High Peak
Ashfield
Arfon
Hodge Hill
Bradford East
Redcar
Scunthorpe
Easington
Hartlepool
Hemsworth
Islwyn
Llanelli
Mansfield
Erewash
Great Grimsby
Luton North
Wrexham
Telford
Torfaen
The Wrekin

New

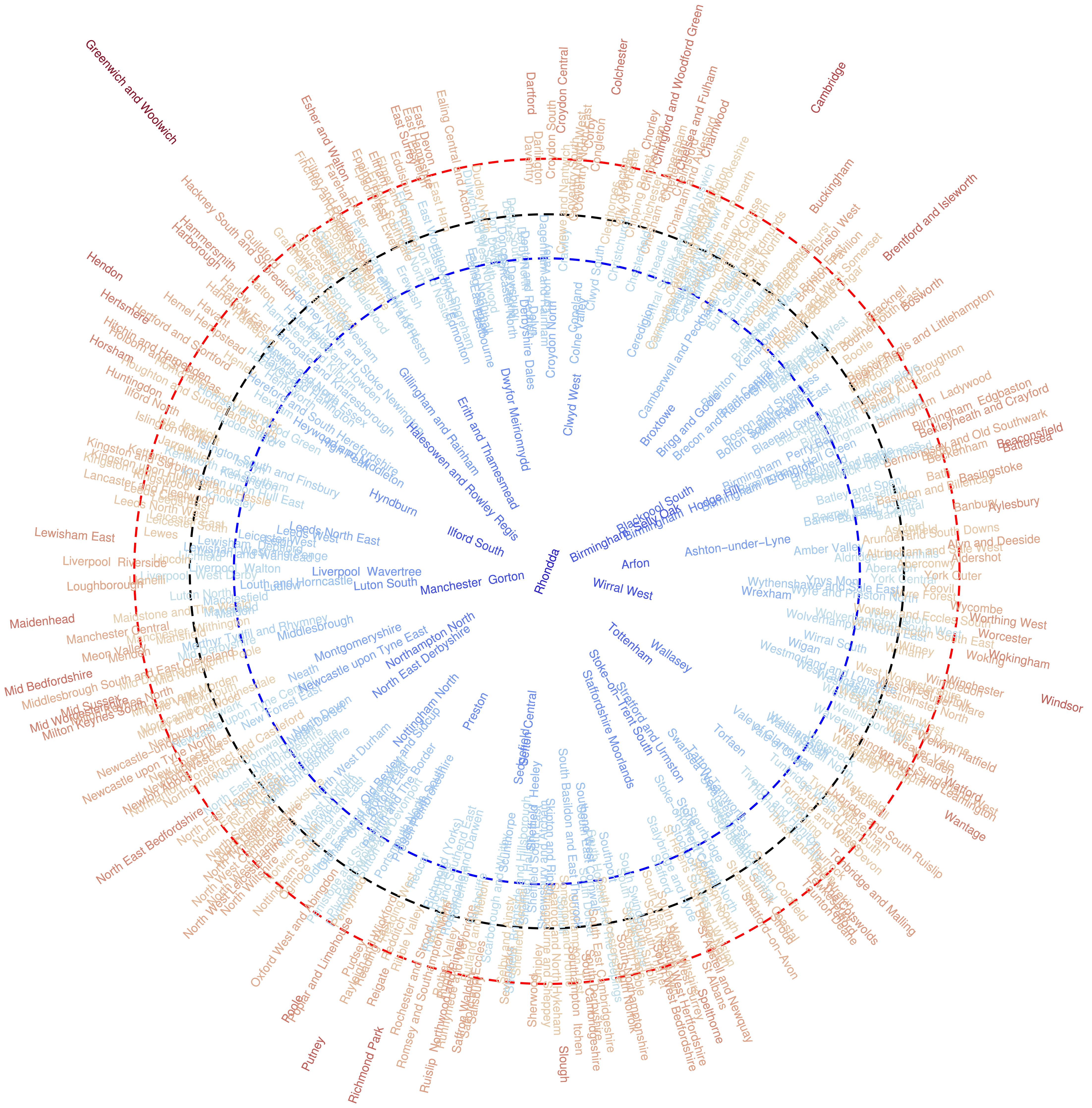
Greenwich and Woolwich
Cambridge
Colchester
Dartford
Croydon Central
Buckingham
Brentford and Isleworth
Hendon
Hertsmere
Horsham
Huntingdon
Lewisham East
Liverpool Riverside
Loughborough
Maidenhead
Mid Bedfordshire
North East Bedfordshire
Windsor
Wantage
Wokingham
Worthing West
Worcester
Aylesbury
Banbury
Battersea
Beaconsfield
Basingstoke
Bosworth
Putney
Richmond Park
Slough
Poole
Rhondda
Manchester Gorton
Ilford South
Arfon
Wirral West
Tottenham
Wallasey
Preston
Sedgefield
Staffordshire Moorlands
Stretford and Urmston
Hyndburn
Dwyfor Meirionnydd
Clwyd West
Broxtowe
Ashton-under-Lyne
Blackpool South
Birmingham, Selly Oak
Gillingham and Rainham
Erith and Thamesmead
Halesowen and Rowley Regis
Luton South
Liverpool Wavertree
Leeds North East
Newcastle upon Tyne East
Northampton North
North East Derbyshire
Nottingham North
Montgomeryshire
Middlesbrough
Tonbridge and Malling
Uxbridge and South Ruislip
Stratford-on-Avon
St Albans
South Cambridgeshire
Oxford West and Abingdon
Poplar and Limehouse
Reigate
Romsey and Southampton North
Ruislip

Old

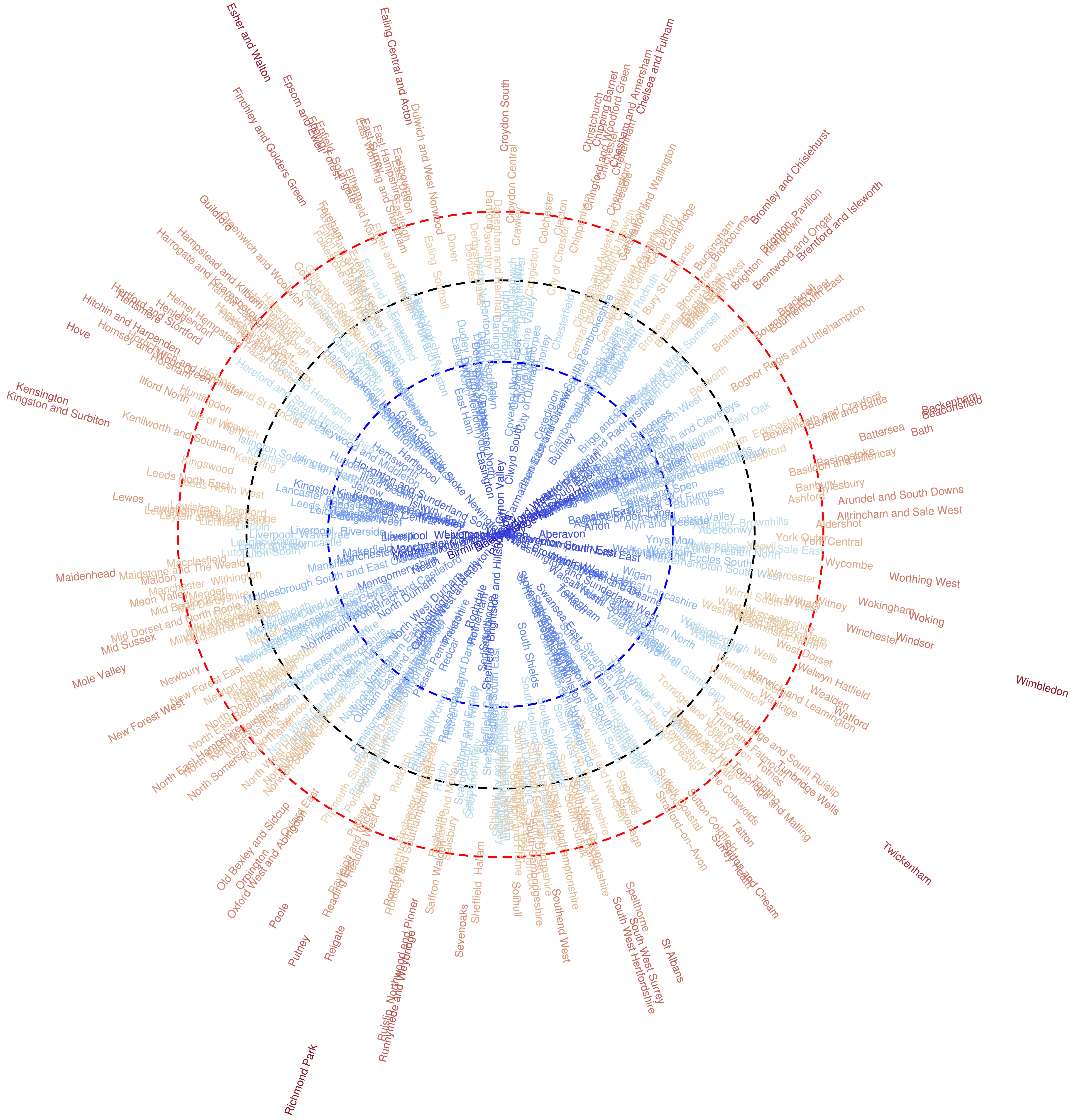

Esher and Walton
Ealing Central and Acton
Epsom and Ewell
Finchley and Golders Green
Dulwich and West Norwood
Croydon South
Croydon Central
Christchurch
Chipping Barnet
Chelsea and Fulham
Bromley and Chislehurst
Brentford and Isleworth
Brentwood and Ongar
Bognor Regis and Littlehampton
Beckenham
Beaconsfield
Bath
Battersea
Arundel and South Downs
Altrincham and Sale West
Aldershot
Wycombe
Worthing West
Wokingham
Woking
Winchester
Windsor
Wimbledon
Welwyn Hatfield
Wealden
Watford
Uxbridge and South Ruislip
Tunbridge Wells
Tonbridge and Malling
Twickenham
Tatton
Sutton and Cheam
Surrey Heath
Stratford-on-Avon
St Albans
South West Surrey
South West Hertfordshire
Spelthorne
Southend West
Solihull
Sheffield, Hallam
Sevenoaks
Saffron Walden
Ruislip, Northwood and Pinner
Runnymede and Weybridge
Richmond Park
Reigate
Putney
Poole
Oxford West and Abingdon
Orpington
Old Bexley and Sidcup
North Somerset
North East Hampshire
New Forest West
Newbury
Mole Valley
Mid Sussex
Mid Dorset and North Poole
Maidenhead
Maidstone and The Weald
Lewes
Leeds North East
Kenilworth and Southam
Kingston and Surbiton
Kensington
Ilford North
Hove
Hitchin and Harpenden
Hertford and Stortford
Hornsey and Wood Green
Harrogate and Knaresborough
Hampstead and Kilburn
Guildford

Order

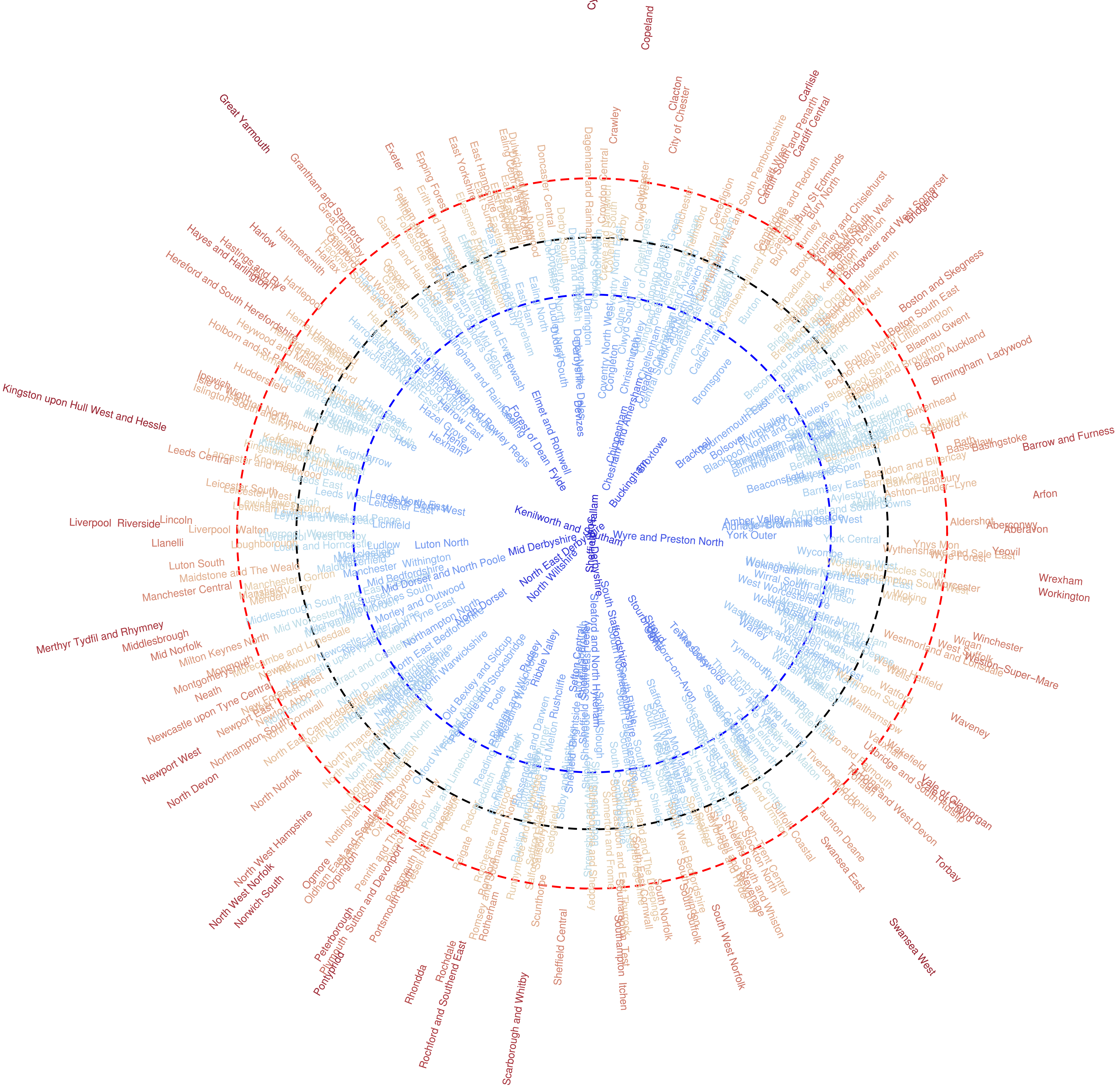

Cynon Valley
Copeland
Clacton
City of Chester
Crawley
Carlisle
Cardiff South and Penarth
Cardiff Central
Great Yarmouth
Grantham and Stamford
Exeter
Epping Forest
Harlow
Hammersmith
Hayes and Harlington
Hastings and Rye
Hereford and South Herefordshire
Kingston upon Hull West and Hessle
Leeds Central
Liverpool Riverside
Lincoln
Llanelli
Luton South
Maidstone and The Weald
Manchester Central
Merthyr Tydfil and Rhymney
Middlesbrough
Mid Norfolk
Milton Keynes North
Neath
Newcastle upon Tyne Central
Newport West
North Devon
North Norfolk
North West Hampshire
North West Norfolk
Norwich South
Ogmore
Peterborough
Pontypridd
Rhondda
Rochdale
Rochford and Southend East
Scarborough and Whitby
Scunthorpe
Sheffield Central
Southampton Itchen
South West Norfolk
Swansea West
Swansea East
Taunton Deane
Torbay
Vale of Glamorgan
Waveney
Wrexham
Workington
Winchester
Westmorland and Lonsdale
Weston-Super-Mare
Aldershot
Aberconwy
Aberavon
Yeovil
Arfon
Barrow and Furness
Basingstoke
Bedford
Birkenhead
Birmingham Ladywood
Bishop Auckland
Blaenau Gwent
Boston and Skegness
Bolton South East
Bridgend
Bridgwater and West Somerset
Bury St Edmunds
Bury North
Burnley
Kenilworth and Southam
Wyre and Preston North
Mid Derbyshire
Mid Dorset and North Poole
North East Derbyshire
North Wiltshire
North Dorset
Elmet and Rothwell
Forest of Dean
Fylde
Chesham and Amersham
Chippenham
Buckingham
Broxtowe
Bracknell
Devizes
Derbyshire Dales
Bromsgrove
Luton North
York Outer
Amber Valley
Aldridge-Brownhills
Ribble Valley
Poole
Stroud
Stourbridge
Tewkesbury
Stratford-on-Avon
The Cotswolds
South Staffordshire
Seaford and North Hykeham
Hexham
Henley
Hazel Grove
Harrow East
Hove
Keighley
Leeds North East
Leeds North West
Leicester East
Lichfield
Ludlow
Withington
Manchester Gorton
Mid Bedfordshire
Morley and Outwood
Northampton North
North East Bedfordshire
North Warwickshire
Old Bexley and Sidcup
Rushcliffe
Erewash
Tynemouth
Wirral South
Wokingham
Wycombe
York Central
Arundel and South Downs
Aylesbury
Barnsley East
Beaconsfield
Calder Valley
Burton
Christchurch
Congleton
Coventry North West
Dudley South
Ealing North

Other Crime

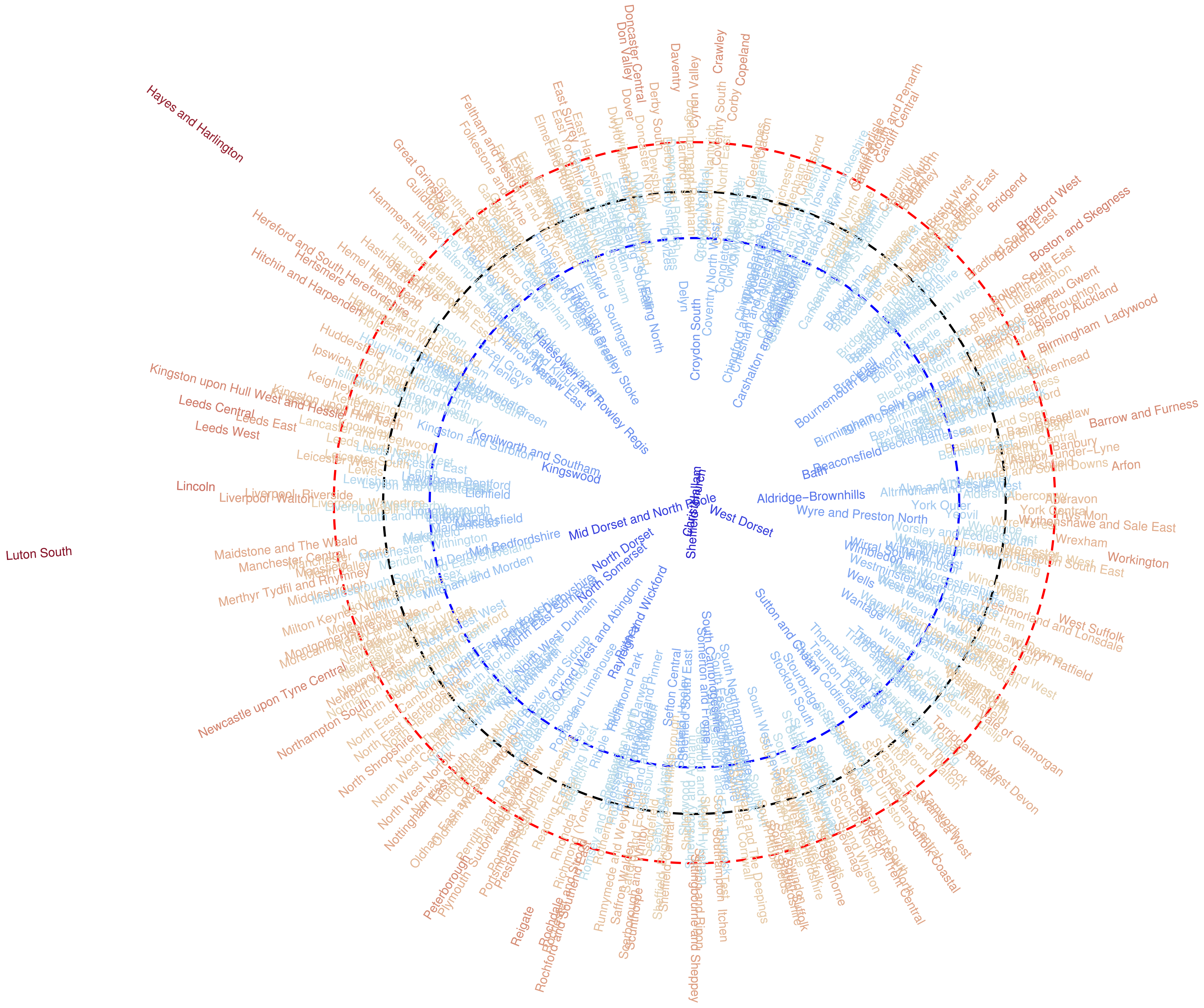

Hayes and Harlington
Luton South
Lincoln
Leeds Central
Leeds East
Leeds West
Kingston upon Hull West and Hessle
Newcastle upon Tyne Central
Northampton South
Hitchin and Harpenden
Hereford and South Herefordshire
Doncaster Central
Don Valley
Daventry
Crawley
Corby
Copeland
Cynon Valley
Cardiff South and Penarth
Cardiff Central
Bridgend
Bradford West
Bradford East
Boston and Skegness
Birmingham Ladywood
Bishop Auckland
Birkenhead
Barrow and Furness
Arfon
Workington
Wrexham
Wythenshawe and Sale East
West Suffolk
Westmorland and Lonsdale
Vale of Glamorgan
Torridge and West Devon
Suffolk Coastal
Reigate
Rochdale
Peterborough
Sheffield, Hallam
West Dorset
Aldridge-Brownhills
Wyre and Preston North
Mid Dorset and North Poole
North Dorset
North Somerset
Mid Bedfordshire
Kingswood
Kenilworth and Southam
Halesowen and Rowley Regis
Croydon South
Carshalton and Wallington
Bournemouth East
Beaconsfield
Bath
Sutton and Cheam
Rayleigh and Wickford
Oxford West and Abingdon
Merthyr Tydfil and Rhymney
Middlesbrough
Maidstone and The Weald
Manchester Central
Liverpool, Riverside
Liverpool, Walton
Great Grimsby
Guildford
Hammersmith
Feltham and Heston
Folkestone and Hythe

Other Theft

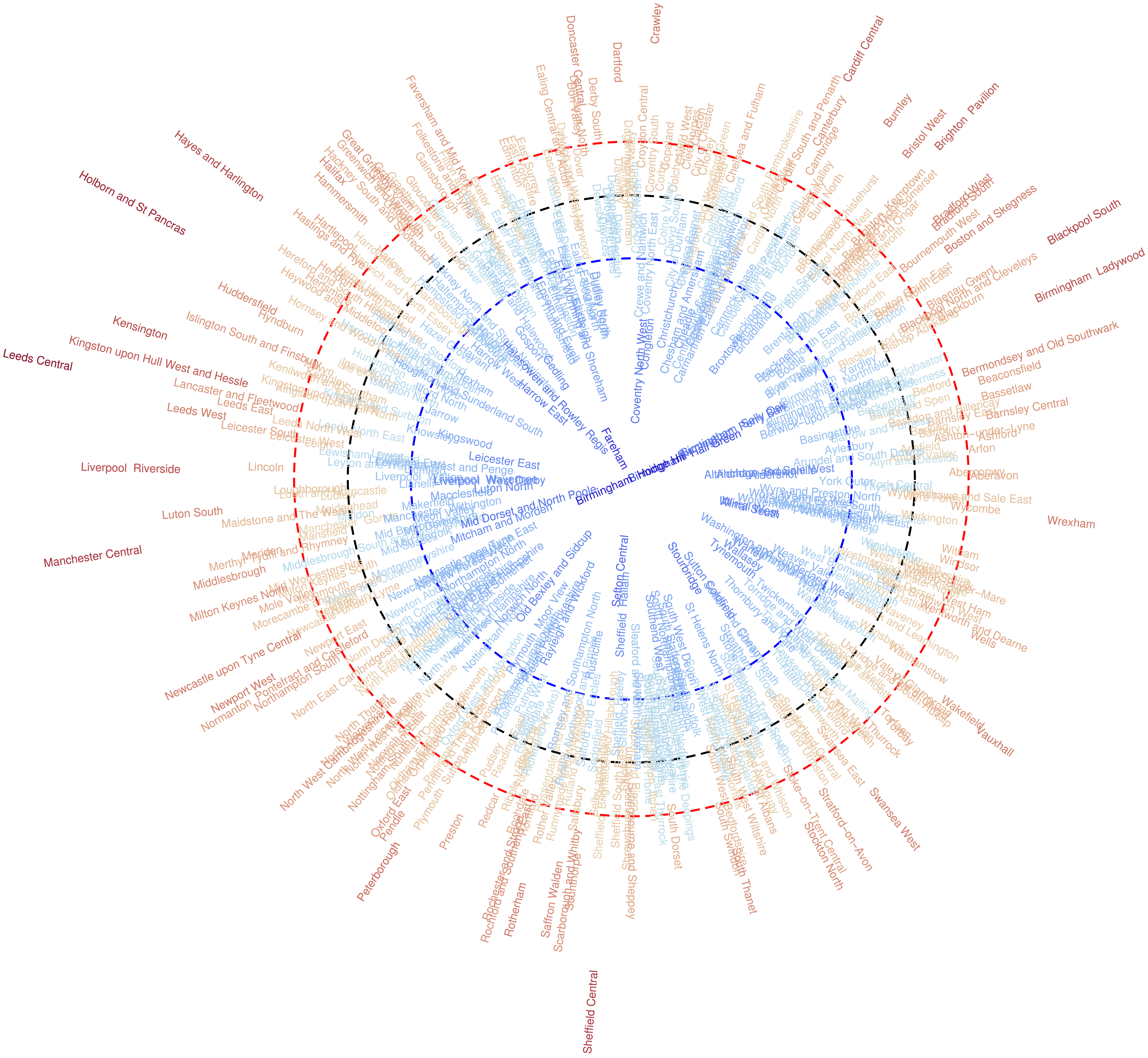
Crawley
Dartford
Cardiff Central
Burnley
Bristol West
Brighton Pavilion
Hayes and Harlington
Holborn and St Pancras
Blackpool South
Birmingham Ladywood
Kensington
Leeds Central
Huddersfield
Kingston upon Hull West and Hessle
Bermondsey and Old Southwark
Leeds West
Liverpool Riverside
Lincoln
Luton South
Manchester Central
Wrexham
Newcastle upon Tyne Central
Newport West
Wakefield
Vauxhall
Swansea West
Peterborough
Preston
Rotherham
Saffron Walden
Sheffield Central
Stockton North
Stratford-on-Avon
South Thanet

Robbery

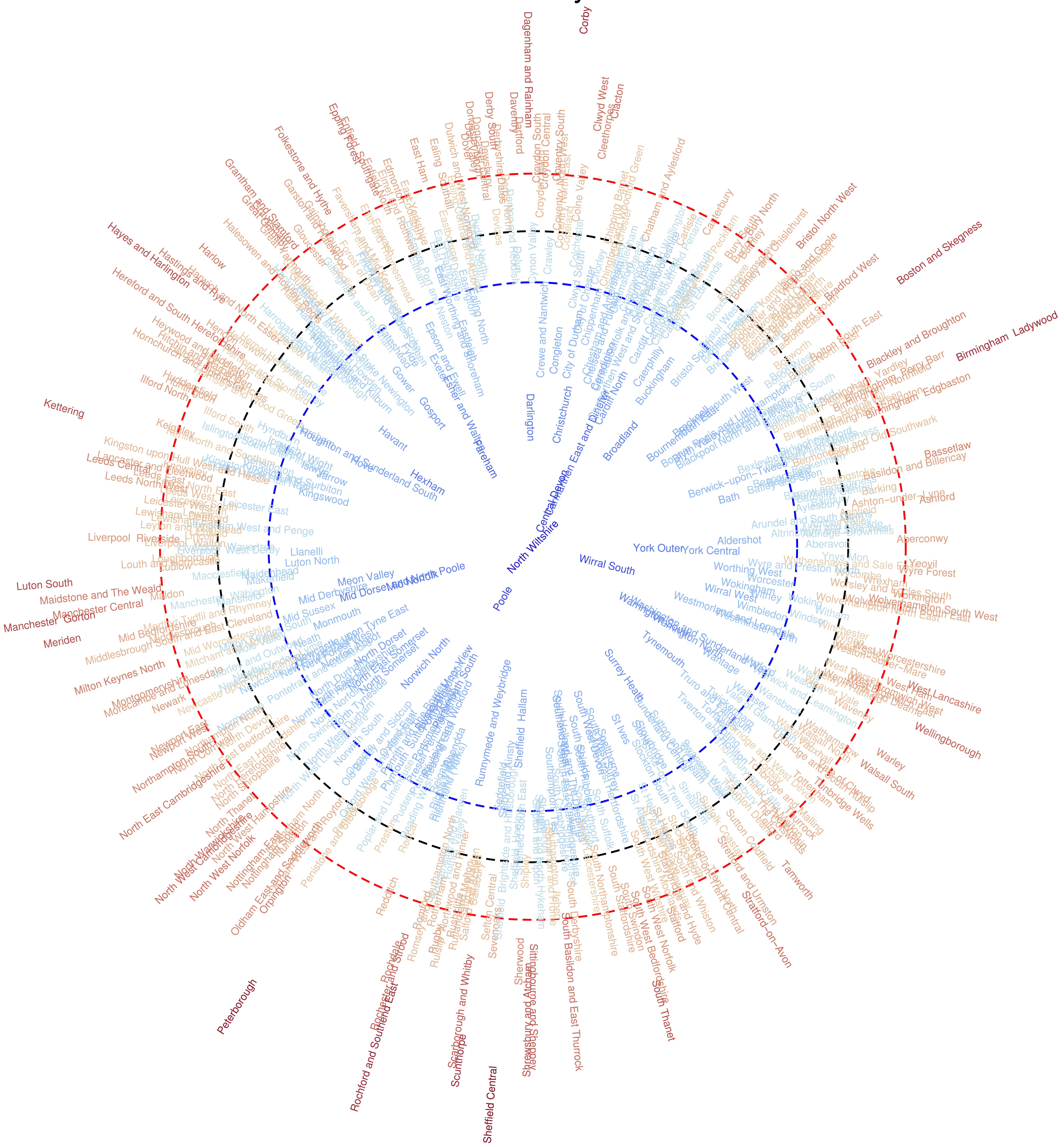

Corby
Dagenham and Rainham
Clwyd West
Clacton
Cleethorpes
Boston and Skegness
Birmingham Ladywood
Bristol North West
Kettering
Luton South
Maidstone and The Weald
Manchester Central
Manchester Gorton
Meriden
Milton Keynes North
North East Cambridgeshire
Hayes and Harlington
Hastings and Rye
Harlow
Hereford and South Herefordshire
Folkestone and Hythe
Epping Forest
Peterborough
Rochford and Southend East
Rochester and Strood
Scarborough and Whitby
Scunthorpe
Sheffield Central
Sittingbourne and Sheppey
Shrewsbury and Atcham
South Basildon and East Thurrock
South Thanet
Stratford-on-Avon
Tamworth
Wellingborough
West Lancashire
Walsall South
Warley
Aberconwy
Ashford
Basildon and Billericay
Bassetlaw
Birmingham Edgbaston
Blackley and Broughton
Bradford West
Yeovil
Wyre Forest
Darlington
Christchurch
Broadland
York Outer
York Central
Wirral South
North Wiltshire
Poole
Havant
Gosport
Hexham
Fareham
Liverpool Riverside
Leeds Central

Semi Detached

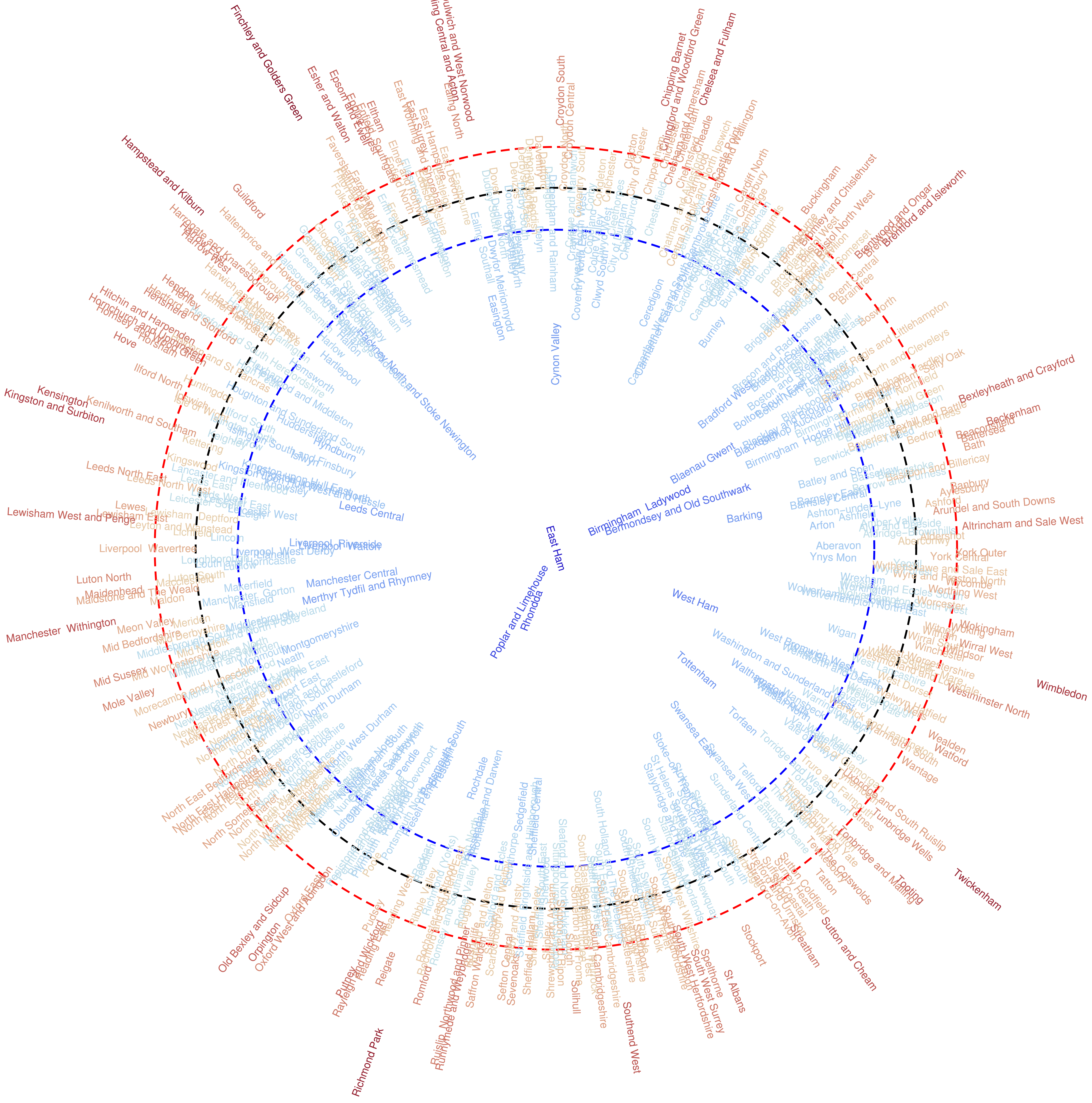

Shoplifting

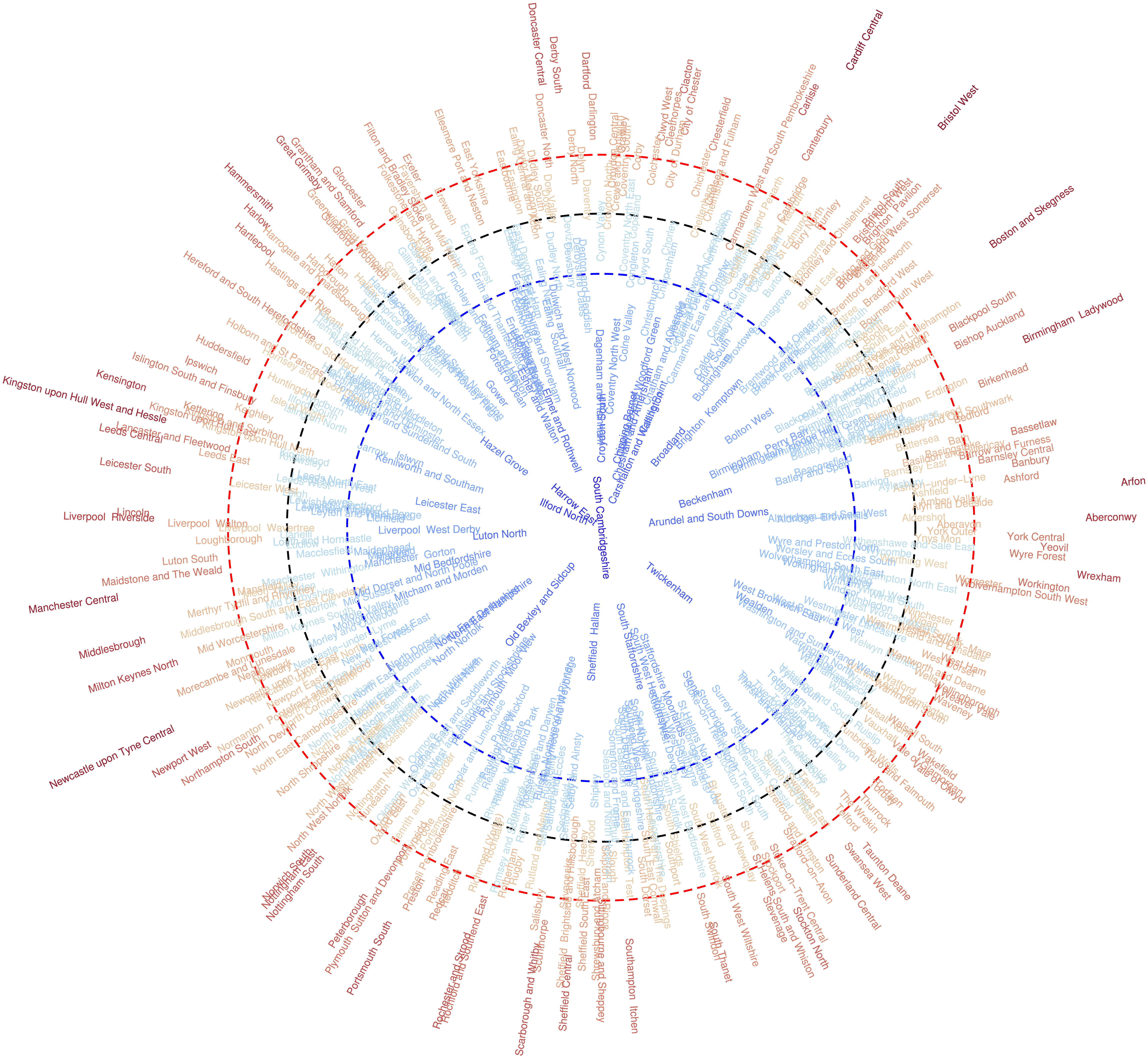

Terraced

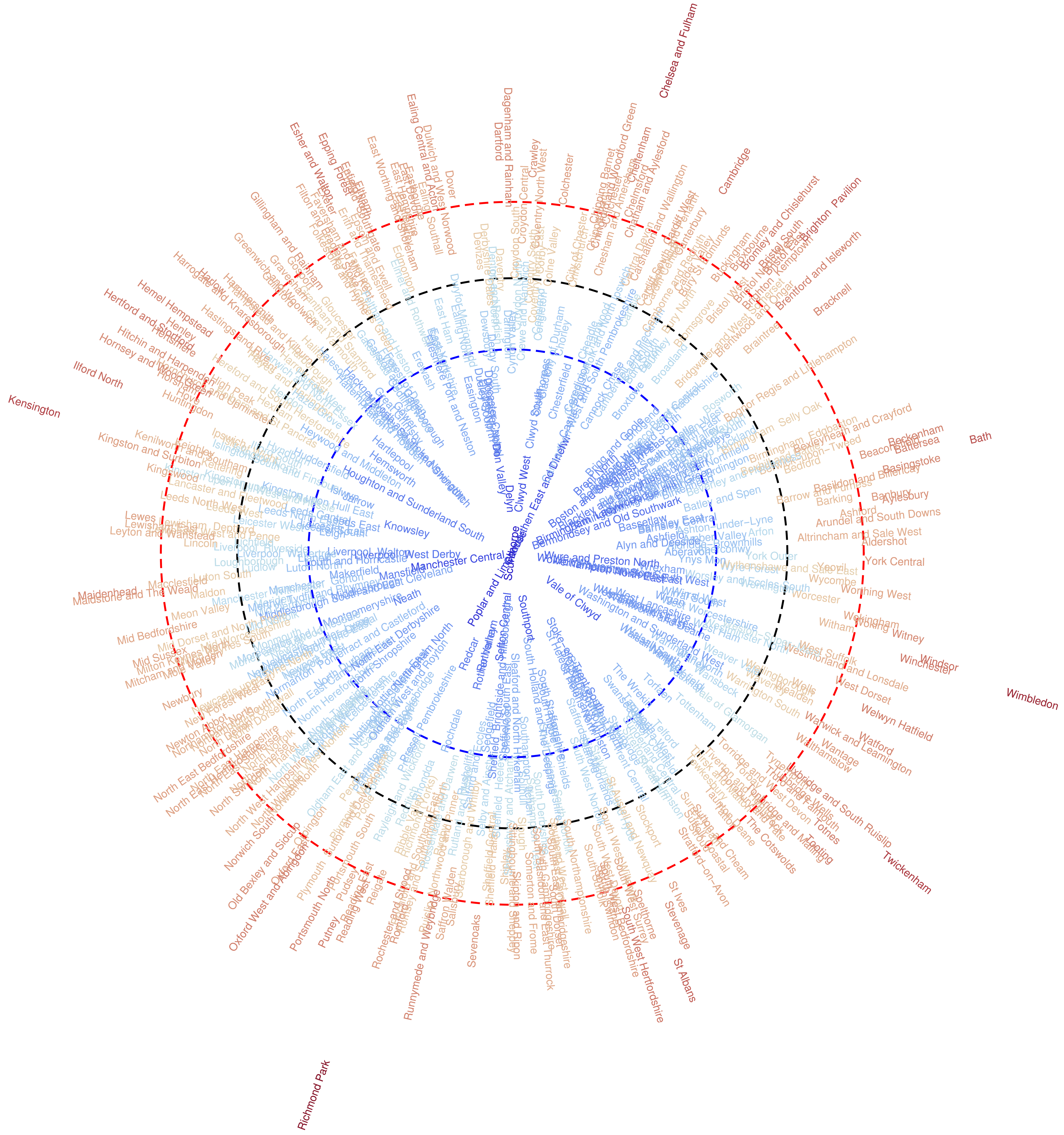

Chelsea and Fulham
Kensington
Richmond Park
Wimbledon
Twickenham
Bath
St Albans
Cambridge
Brighton, Pavilion
Bracknell
Beckenham
Battersea
Basingstoke
Aylesbury
Aldershot
York Central
Worthing West
Wokingham
Witney
Windsor
Winchester
Welwyn Hatfield
Watford
Wantage
Warwick and Leamington
Uxbridge and South Ruislip
Tooting
Stevenage
St Ives
South West Hertfordshire
Sevenoaks
Runnymede and Weybridge
Reading West
Putney
Portsmouth North
Oxford West and Abingdon
Old Bexley and Sidcup
Norwich South
North West Hampshire
North East Bedfordshire
Newbury
Mitcham and Morden
Mid Sussex
Mid Bedfordshire
Maidstone and The Weald
Maidenhead
Macclesfield
Leyton and Wanstead
Lewes
Kingston and Surbiton
Ilford North
Hornsey and Wood Green
Hitchin and Harpenden
Hertford and Stortford
Hemel Hempstead
Henley
Harrogate and Knaresborough
Gillingham and Rainham
Esher and Walton
Epping Forest
Ealing Central and Acton
Dulwich and West Norwood
Dover
Dagenham and Rainham
Dartford
Crawley
Colchester
Cheltenham
Carshalton and Wallington
Bromley and Chislehurst
Brentford and Isleworth
Manchester Central
Poplar and Limehouse
Knowsley
Houghton and Sunderland South
Bermondsey and Old Southwark
Vale of Clwyd
Southport
Clwyd West
Delyn
Liverpool, West Derby
Washington and Sunderland West
Redcar
Pembrokeshire
Rochdale
Tottenham
Telford
The Wrekin
Hartlepool
Hemsworth
Easington
Chesterfield
Broxtowe
Batley and Spen
Wrexham
York Outer
Worcester
Wycombe
Witham
Wells
West Dorset
Walthamstow
Stratford-on-Avon
Stockport
Somerton and Frome
Saffron Walden
Reigate
Pudsey
Plymouth, Sutton and Devonport
Newton Abbot
Meon Valley
Maldon
Lincoln
Ludlow
Loughborough
Luton South
Kingswood
Kenilworth and Southam
Ipswich
Huntingdon
Hereford and South Herefordshire
Hastings and Rye
Gravesham
Erith and Thamesmead
Devizes
Derbyshire Dales
Colne Valley
Chippenham
Chesham and Amersham
Chelmsford
Bury St Edmunds
Buckingham
Braintree
Bognor Regis and Littlehampton
Selly Oak
Basildon and Billericay
Barking
Banbury
Ashford
Arundel and South Downs
Altrincham and Sale West
Yeovil
Worsley and Eccles South

Theft From Person

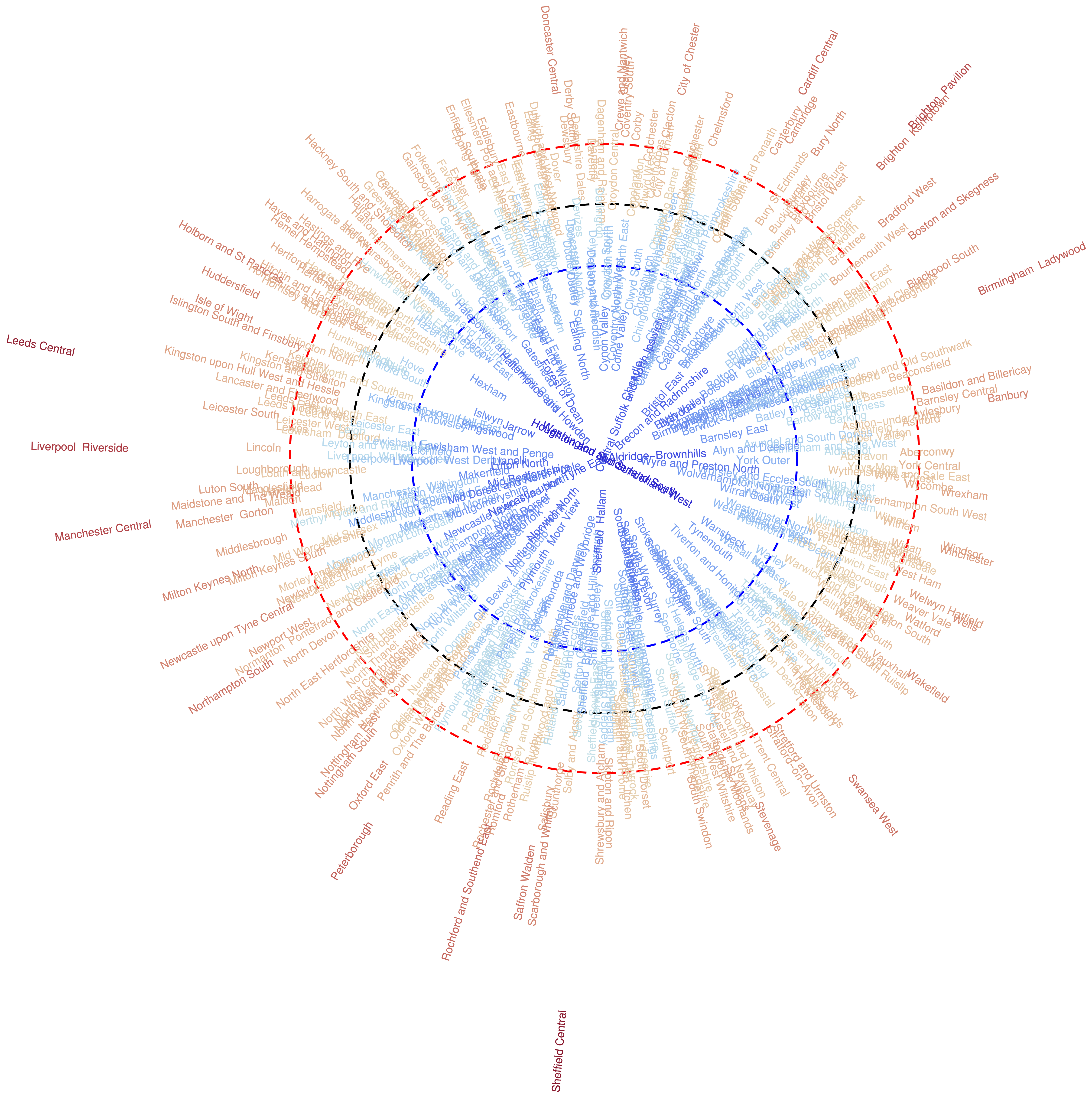

Vehicle Crime

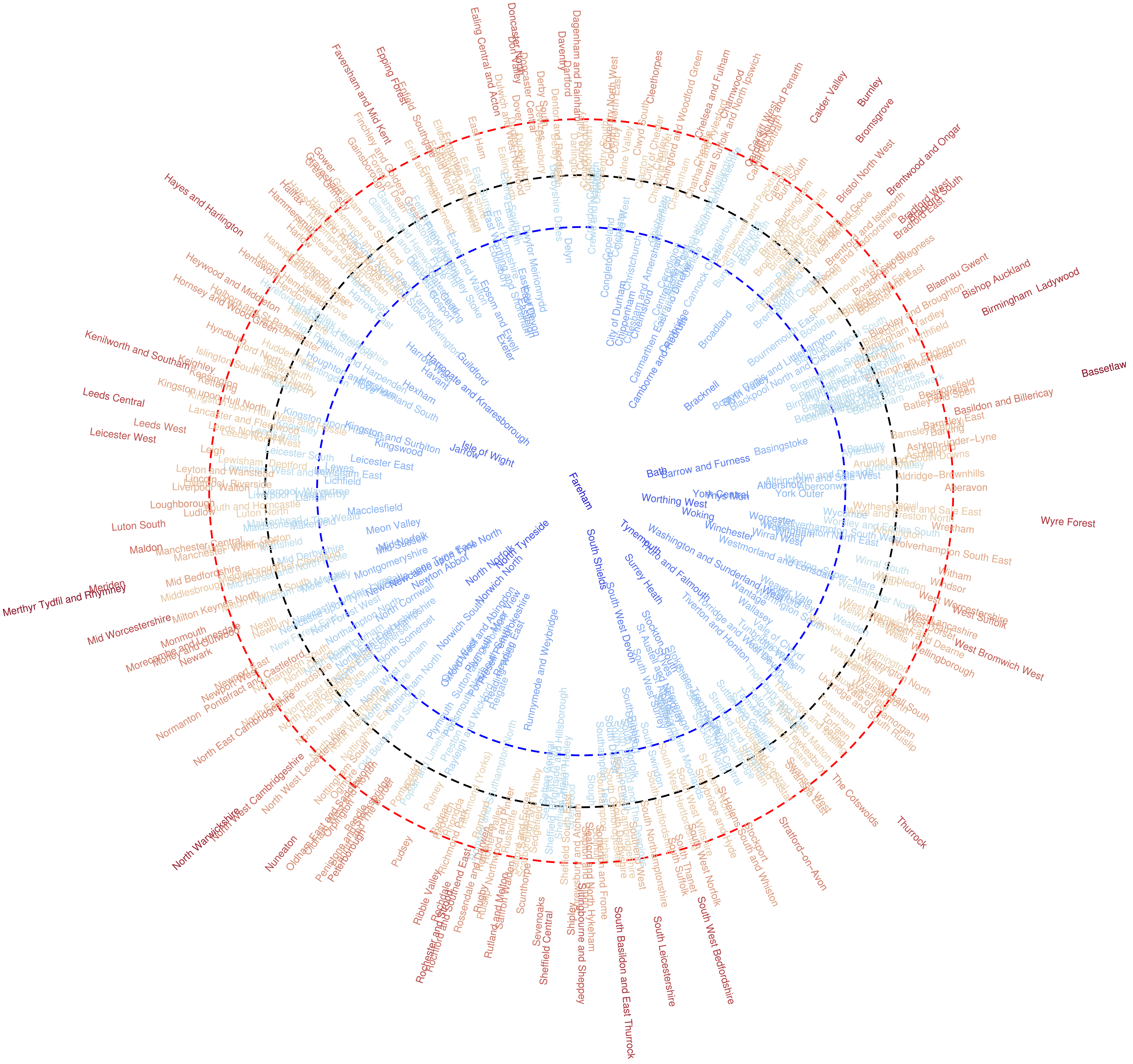

Violence

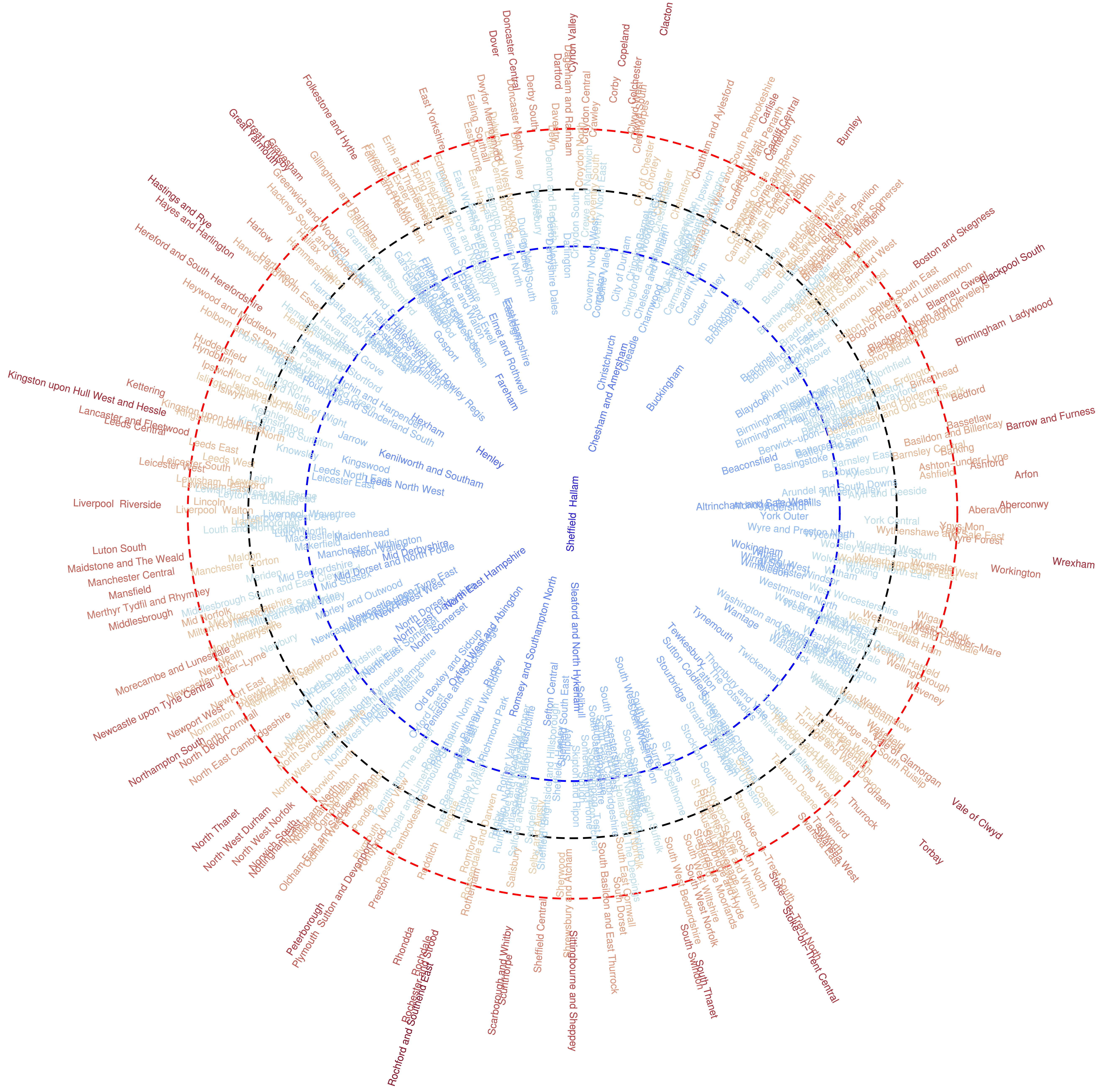

Weapons

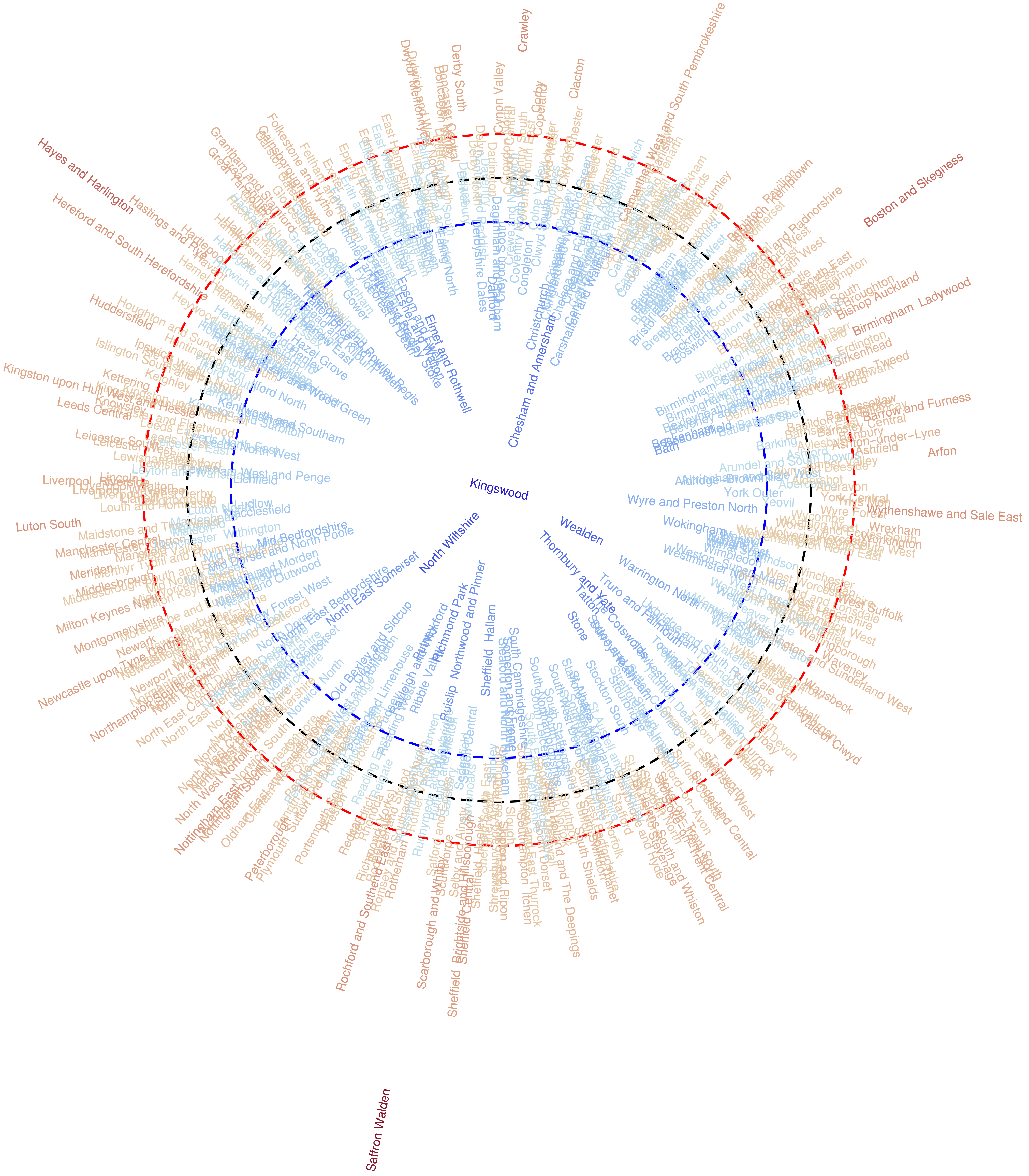
Crawley
Clacton
Derby South
Hayes and Harlington
Hastings and Rye
Hereford and South Herefordshire
Huddersfield
Kingston upon Hull West and Hessle
Leeds Central
Liverpool, Riverside
Luton South
Meriden
Milton Keynes North
Newcastle upon Tyne Central
Northampton North
Peterborough
Rochford and Southend East
Scarborough and Whitby
Sheffield, Brightside and Hillsborough
Sheffield Central
Saffron Walden
Boston and Skegness
Carmarthen West and South Pembrokeshire
Bishop Auckland
Birmingham, Ladywood
Birkenhead
Barrow and Furness
Arfon
Wythenshawe and Sale East
Wrexham
Suffolk
Sunderland West
Vale of Clwyd
Stoke-on-Trent Central
Kingswood
Chesham and Amersham
Elmet and Rothwell
Wealden
North Wiltshire
North East Somerset
Mid Bedfordshire
Wyre and Preston North
Thornbury and Yate
Truro and Falmouth
Warrington North
Wokingham
Stone
Tatton
Sheffield, Hallam
South Cambridgeshire
Richmond Park
Ruislip, Northwood and Pinner
Old Bexley and Sidcup
North East Bedfordshire
Kenilworth and Southam
Beckenham
Bath
York Outer
Yeovil
Gower
Hazel Grove
Derbyshire Dales
Congleton
Christchurch
Lichfield
Ludlow
Macclesfield
Mid Dorset and North Poole
Aldridge-Brownhills
Arundel and South Downs
Westminster North
Wimbledon
