# Supplementary figures and images for: Unveiling relationships between crime and property in England and Wales via density scale-adjusted metrics and network tools
Source: PLoS One. 2018 Feb 22;13(2):e0192931. doi: 10.1371/journal.pone.0192931 (PMC5823401; doi:10.1371/journal.pone.0192931)

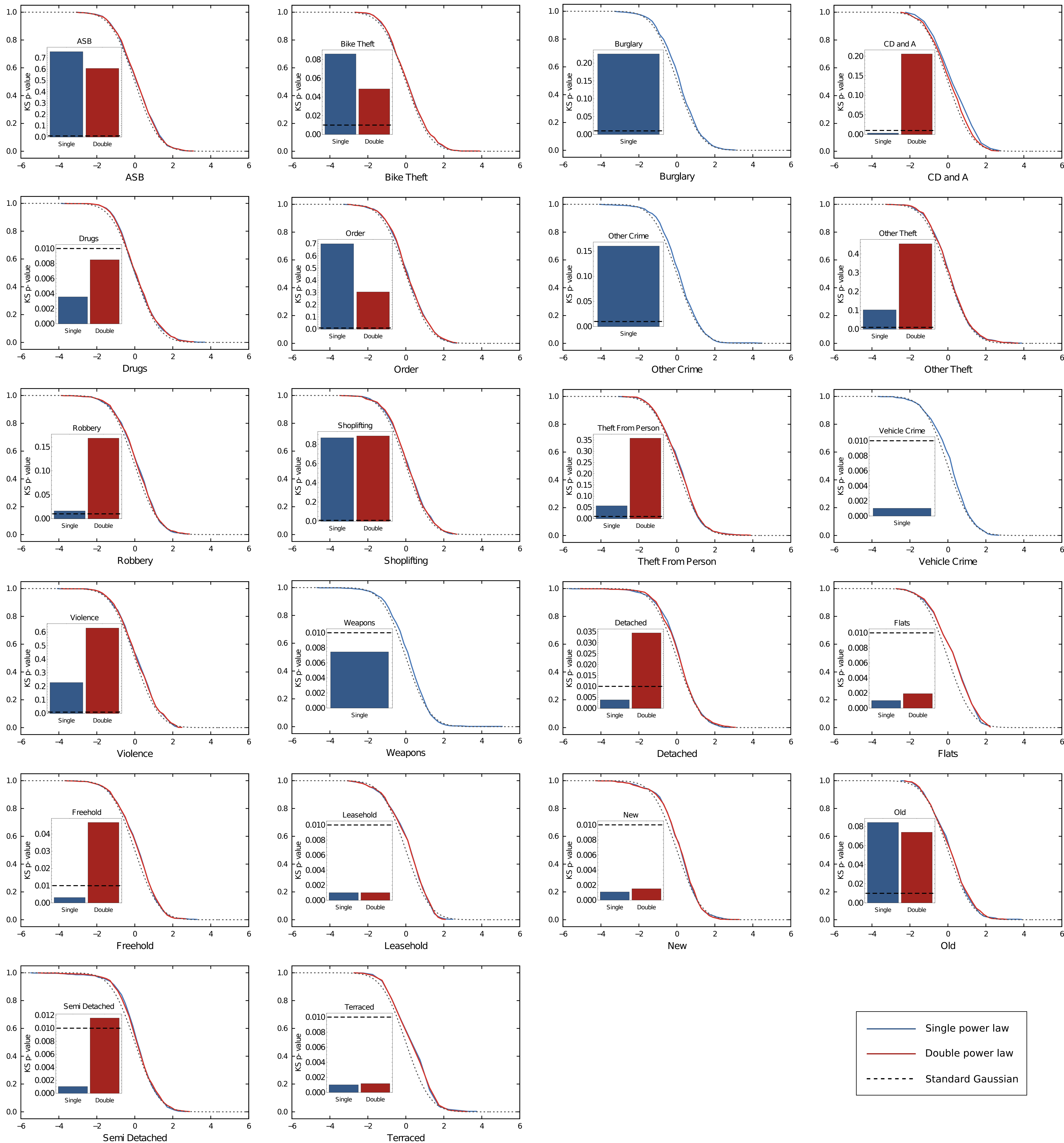

Density Scale-Adjusted Metrics (SAM)

Supplement: S1 Fig — The panels show the cumulative distributions of the normalized DSAMs (that is, after subtracting its mean and dividing by its standard deviation) for each crime and property type. For DSAMs in which the double power-law model is a better fit, we show the distributions of the normalized DSAM defined with the single power-law model (blue lines) and with double power-law model (red lines). The insets in each plot show the p-values of the Kolmogorov-Smirnov normality test, where the horizontal dashed lines indicate the 0.01 confidence level threshold. We note that the normality of the DSAMs is achieved for criminal damage and arson (CD and A), detached, freehold and semi detached only when defining the DSAM with the double power-law model. We further observe that double power-law usually produces higher p-values. (PDF) [file pone.0192931.s003.pdf]

DSAM standard deviation

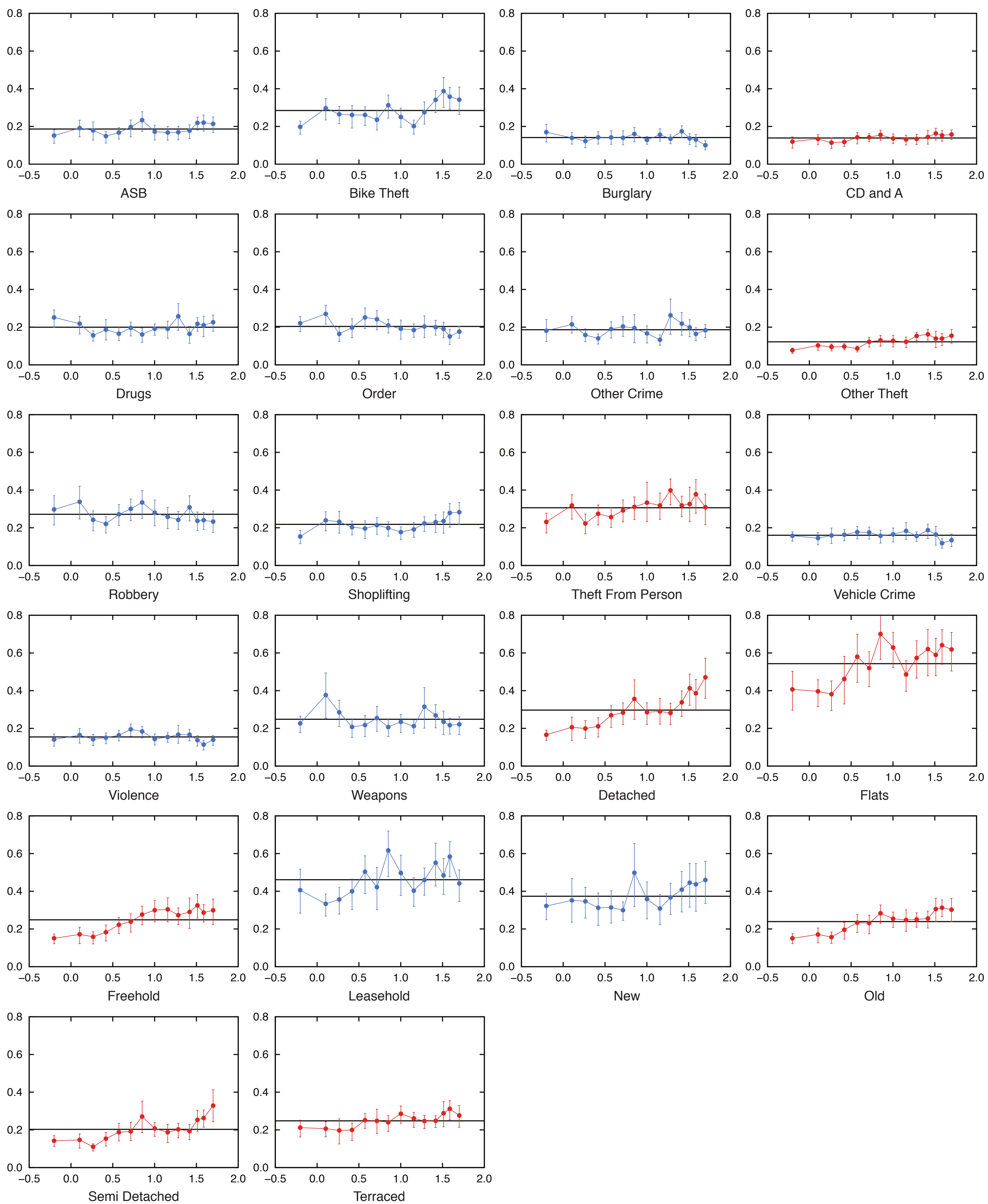

Log(Population density)

Supplement: S2 Fig — Each panel shows the standard deviation of the DSAM evaluated in 15 equally-spaced windows over population density on a logarithmic scale. The error bars are 99% bootstrap confidence intervals and the horizontal lines are the average values of standard deviations for each indicator. The plots with blue markers are the ones for which no significant increasing trend is observed between standard deviation and population density via linear regressions, whereas the plots with red markers show the indicators for which this relationship has a significant linear increasing trend. Despite the statistical significance of some linear coefficients (9 out of 22), we observe that the majority of the relationships do not show large deviations from the horizontal plateau defined by the mean of the standard deviation. (PDF) [file pone.0192931.s004.pdf]

DSAM standard deviation growth rate  
(Units of DSAM) / (Log(p/ha))

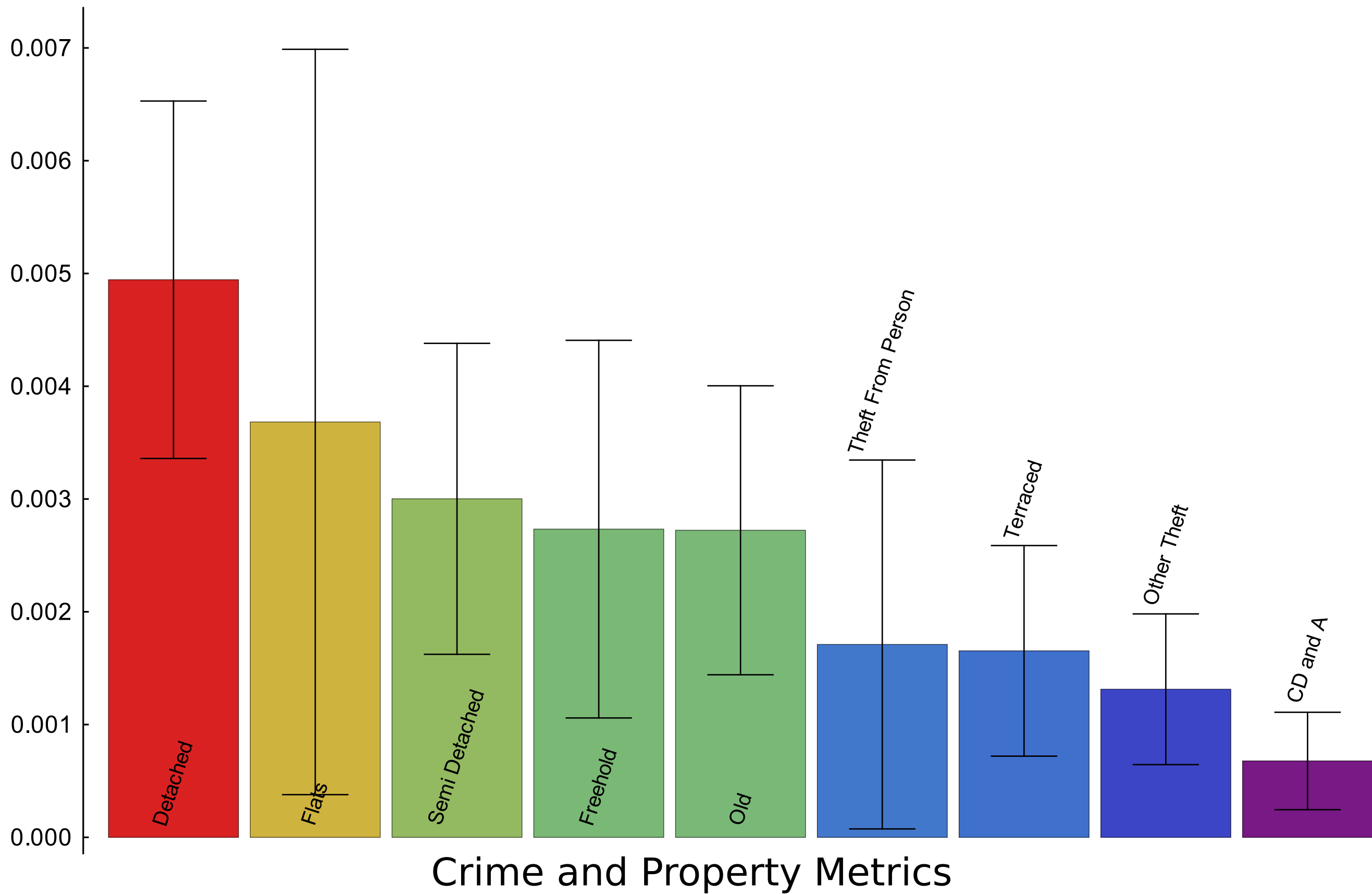

Supplement: S3 Fig — Linear coefficients of the linear regressions between DSAM standard deviation and log of population density that are statistically significant. Despite the significance of the increasing trends for 9 out of 22 metrics, we note that the growth rates are very small (up to 0.005 units of DSAM per log[p/ha]). (PDF) [file pone.0192931.s005.pdf]

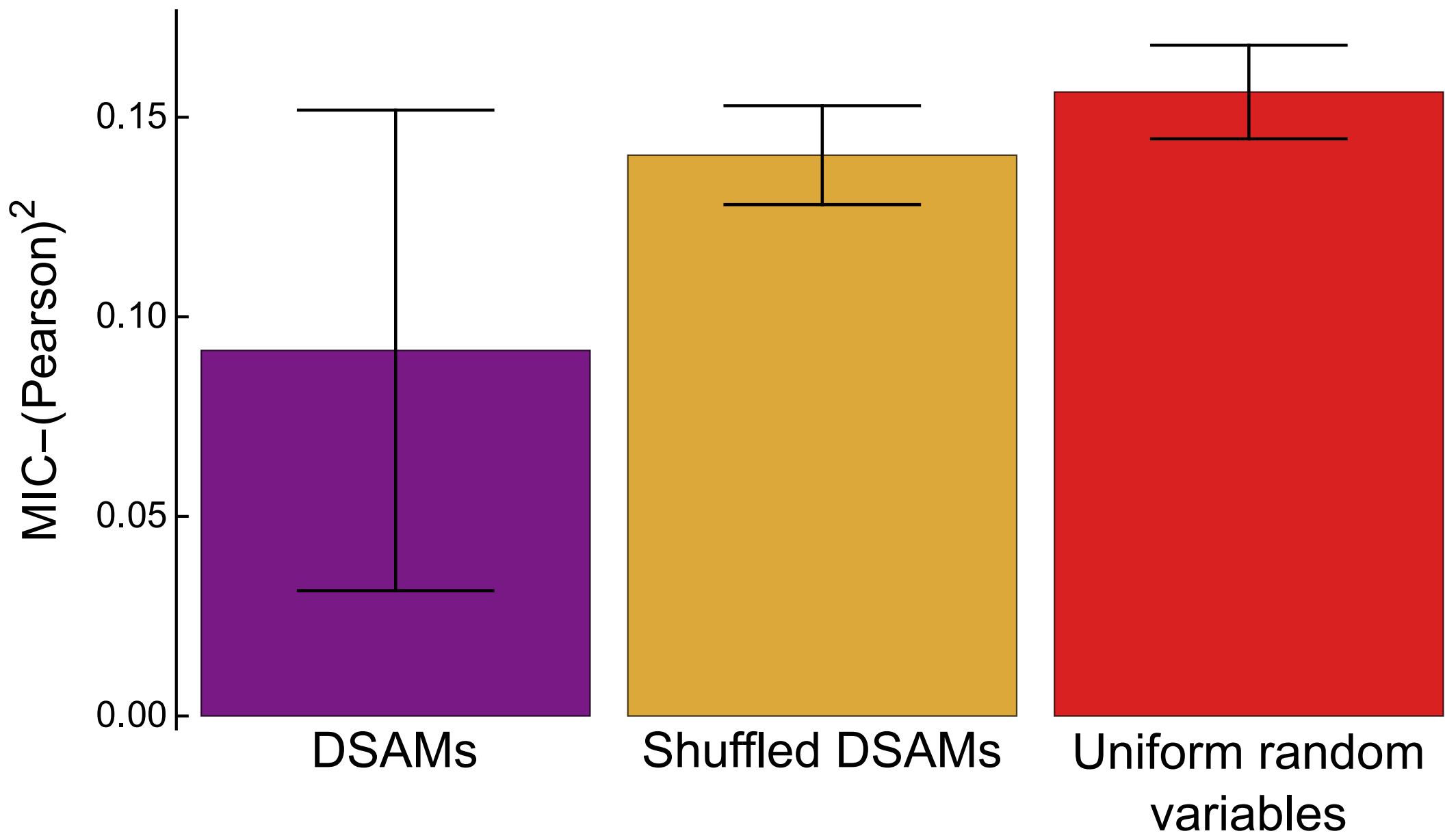

Supplement: S4 Fig — The bar plot show the average value of the difference between the maximal information coefficient (MIC) [47] and square of the Pearson linear correlation over over all unique pairs of DSAM types (MIC-Pearson2), for the DSAMs values after shuffling their values among constituencies, and for uniform random numbers (sample size is equal to the DSAM case). Error bars are one standard deviation of the quantity (MIC-Pearson2). We note that the average value for original DSAMs is small and fully explained by chance. Thus, we can assume that correlations among the DSAMs are linear. (PDF) [file pone.0192931.s006.pdf]

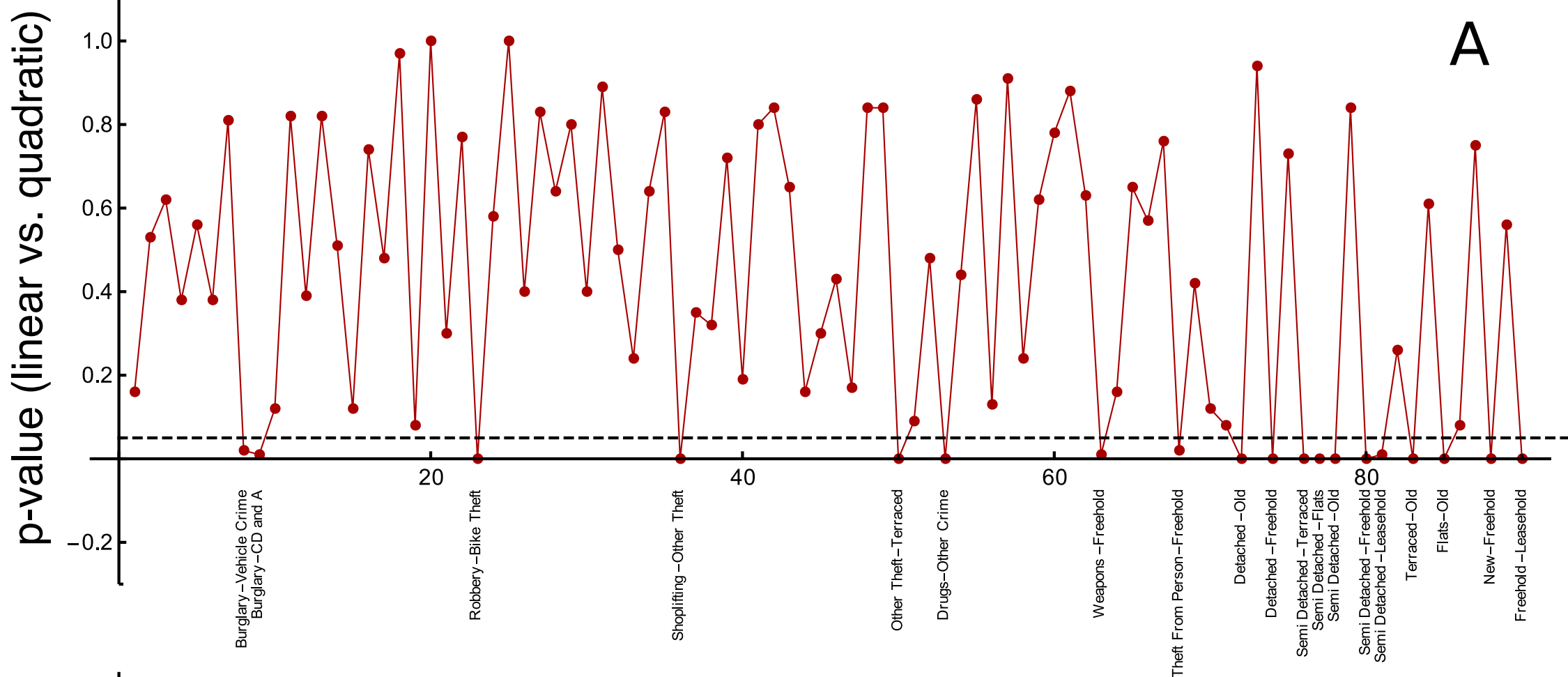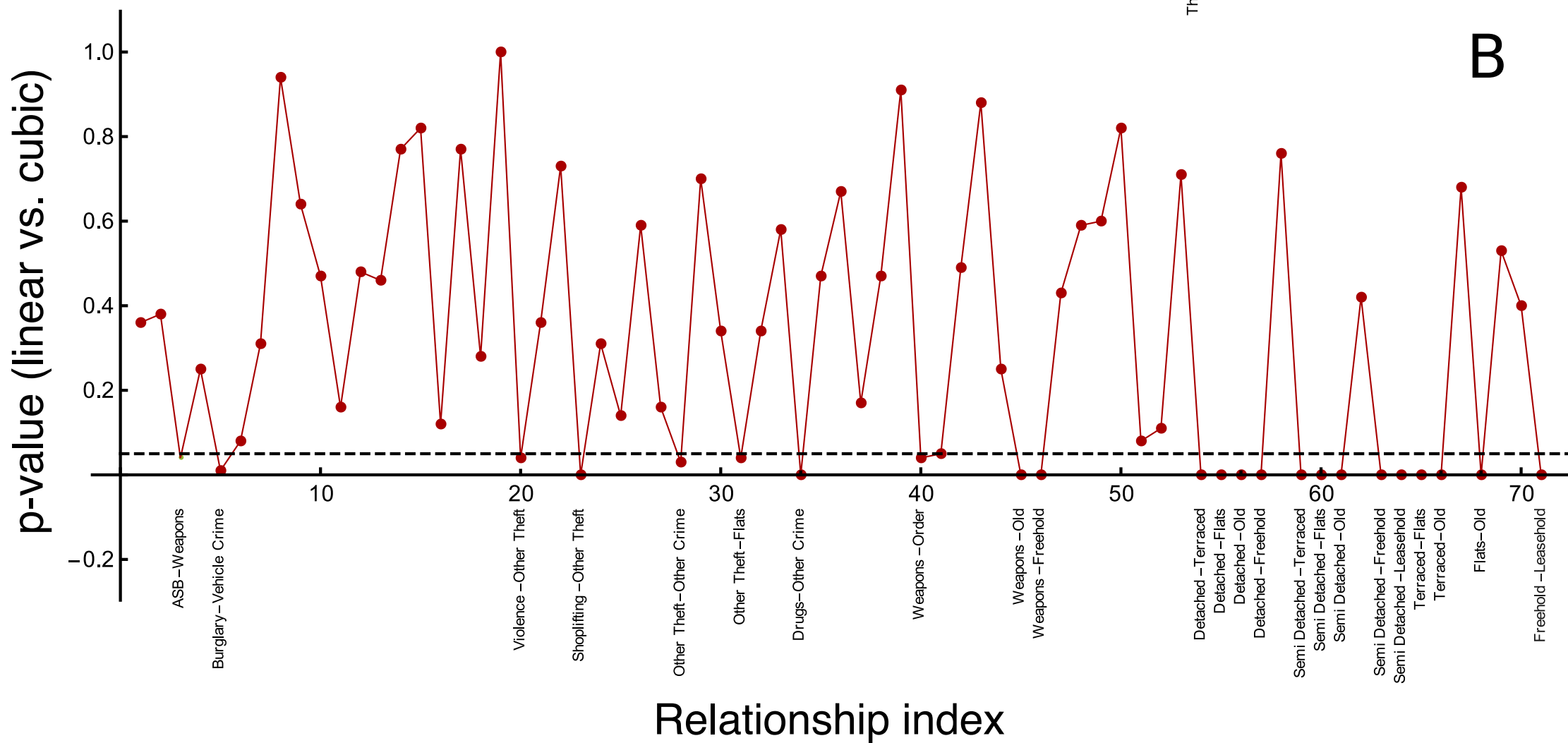

Supplement: S5 Fig — (A) Each dot corresponds to the p-value of the two-sample bootstrap test (at 95% confidence)for the equality of mean values of the Bayesian information criterion (BIC) obtained by adjusting the DSAMs pairwise relationships with a linear and a quadratic model. We have bootstrapped the BIC values over 100 realizations and considered only the pairwise relationships in which the BIC for quadratic model is smaller than the one obtained for the linear model. Among the 231 possible relationships, the BIC of the quadratic model is smaller than BIC of the linear in 90 cases. However, the difference between the BIC values is significant only in 19 cases (indicated in the plot), that is, only in ≈8% of all relationships. (B) The same analysis comparing the linear model with the cubic model. In this case, the BIC of the cubic model is smaller than BIC of the linear in 71 cases, but in only 23 there is significant difference, only in ≈10% of all relationships. (PDF) [file pone.0192931.s007.pdf]
