# Supplementary material for: Unveiling relationships between crime and property in England and Wales via density scale-adjusted metrics and network tools
Source: PLoS One. 2018 Feb 22;13(2):e0192931. doi: 10.1371/journal.pone.0192931 (PMC5823401; doi:10.1371/journal.pone.0192931)
Supplement: S6 Fig — Each gray node (circle) represents a crime or a property type, and the connections indicate the metrics that are negatively (blue) and positively (red) correlated with the particular crime or property type. The edge thickness is proportional to the absolute value of Pearson correlation ρij between the metrics, and the edge label shows the value of ρij. The edges are arranged so that negative correlations are above the gray nodes, and positive ones are below; also, the closer the edge is to the vertical position, the more intense is the correlation/anti-correlation between the metrics. (PDF) [file pone.0192931.s008.pdf]

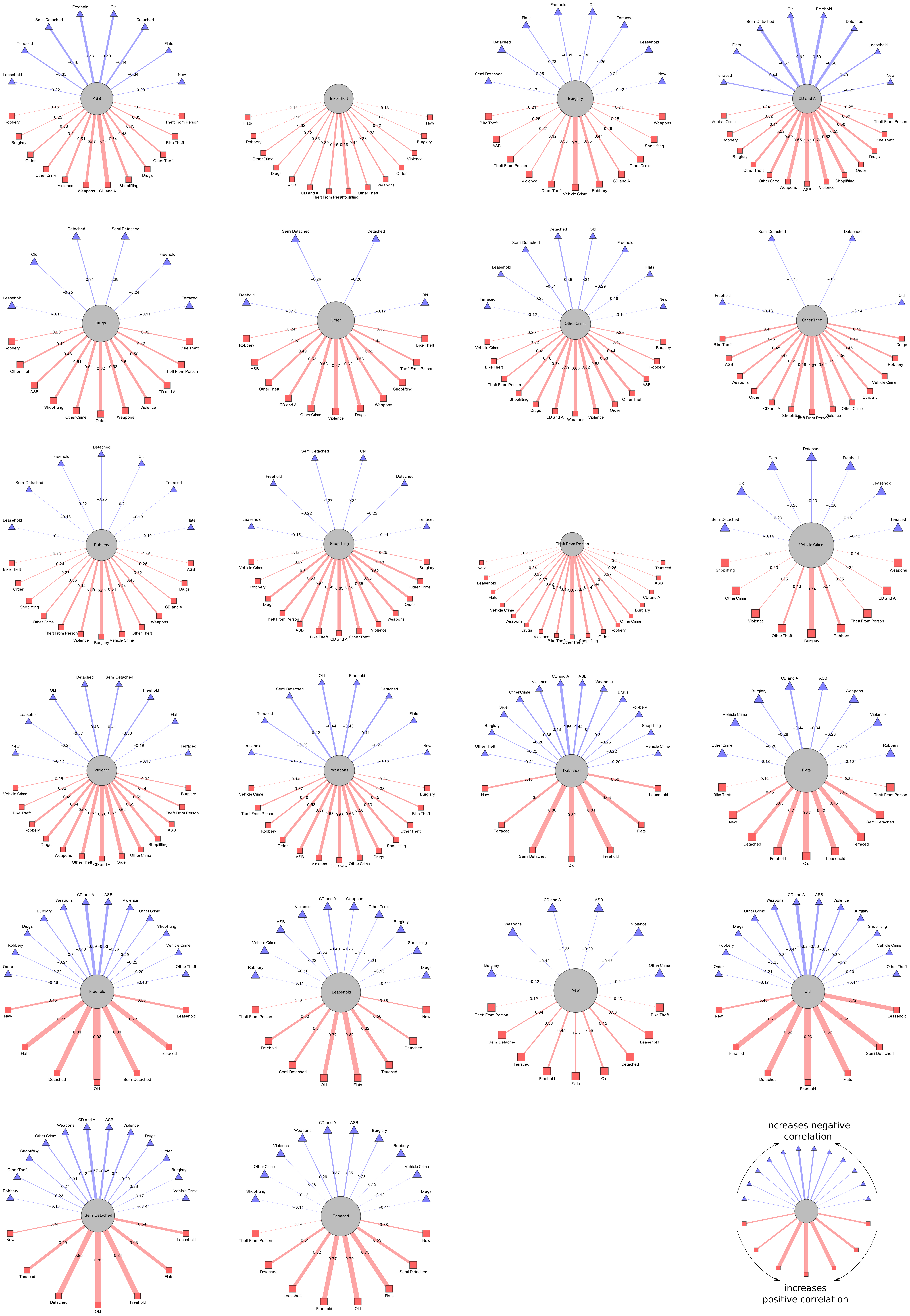
ASB
Bike Theft
Burglary
CD and A
Drugs
Order
Other Crime
Other Theft
Robbery
Shoplifting
Theft From Person
Vehicle Crime
Violence
Weapons
Detached
Flats
Freehold
Leasehold
New
Old
Semi Detached
Terraced
increases negative correlation
increases positive correlation
